# TRPV4 drives macrophage pyroptosis via mitochondrial dysfunction and mtROS-dependent NLRP3 inflammasome activation in acute lung injury

**DOI:** 10.1016/j.redox.2026.104255

**Published:** 2026-06-11

**Authors:** Lan Luo, Xiaofang Yang, Shuyuan Yi, Ziyuan Dong, Kan Wang, Zicheng Zhu, Qian Gao, Jianxue Gao, Yu Jiang, Ming Gong, Hongjia Zhang, Meili Wang, Feilong Hei

**Affiliations:** a Department of Extracorporeal Circulation and Mechanical Circulation Assistants, Center for Cardiac Intensive Care, Beijing Anzhen Hospital, Capital Medical University, No. 2 Anzhen Road, Chaoyang District, Beijing, 100029, China; b Department of Anesthesiology, Sichuan Provincial People's Hospital, School of Medicine, University of Electronic Science and Technology of China, Chengdu, 610072, China; c Beijing Lab for Cardiovascular Precision Medicine, Capital Medical University, Beijing, 100069, China; d Department of Cardiac Surgery, Beijing Anzhen Hospital, Capital Medical University, No. 2 Anzhen Road, Chaoyang District, Beijing, 100029, China; e Department of Physiology and Pathophysiology, School of Basic Medical Sciences, Capital Medical University, Beijing, 100069, China

## Abstract

• Macrophage TRPV4 promotes pyroptosis and aggravates acute lung injury.
• TRPV4-mediated Ca^2+^ influx induces mitochondrial dysfunction and mtROS production.
• mtROS drive NLRP3 inflammasome activation downstream of TRPV4 signaling.

## Graphical abstract

Created in BioRender. Luo, L. (2026) https://BioRender.com/dd42nkh.

**Figure:**
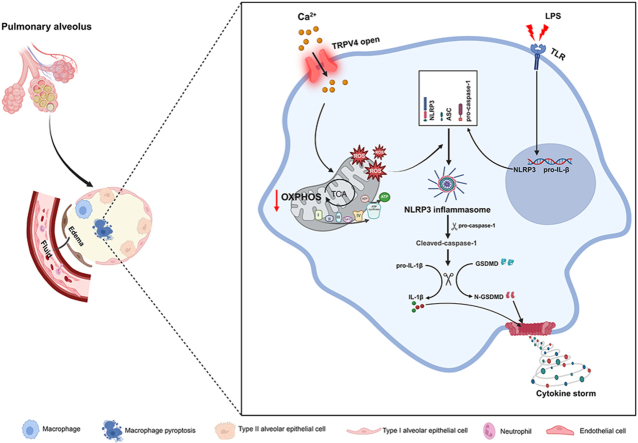

## 1 Introduction

Acute lung injury (ALI) and its severe manifestation, acute respiratory distress syndrome (ARDS), are life-threatening clinical syndromes characterized by overwhelming pulmonary inflammation, diffuse alveolar damage, and increased vascular permeability, frequently culminating in hypoxemic respiratory failure [1,2]. These conditions may arise from a broad spectrum of pulmonary and extrapulmonary insults, including pneumonia, systemic infection, ischemia/reperfusion injury, mechanical ventilation, chemical toxins, and drug-induced lung injury [3,4]. ALI/ARDS typically progresses rapidly, with clinical deterioration occurring within hours to days, and remains associated with high morbidity and mortality. Notably, mortality among patients with severe COVID-19-associated ARDS has been reported to exceed 70%, underscoring the profound global health burden of this syndrome [5,6]. Despite substantial advances in supportive care, such as lung-protective ventilation strategies and prone positioning, effective pharmacological interventions targeting the underlying inflammatory pathology are still lacking [7,8], highlighting an urgent need to identify novel molecular targets.

Pulmonary macrophages represent a key innate immune cell population in the lung and play a central role in sensing danger signals and orchestrating inflammatory responses during ALI [9,10]. Pathogenic or sterile insults activate multiple intracellular inflammatory signaling pathways in macrophages, among which lytic forms of cell death, such as pyroptosis and necroptosis, markedly amplify inflammatory responses and drive disease progression through the release of inflammatory mediators and intracellular components [11]. As a central signaling platform for inflammatory responses induced by pathogen infection and tissue damage, the inflammasome exerts critical regulatory functions in a wide range of inflammation-related diseases [12]. Within the inflammasome complex, NOD-like receptor family pyrin domain-containing 3 (NLRP3) is considered a key sensor driving pulmonary inflammation. Upon activation, the adaptor protein apoptosis-associated speck-like protein containing a CARD (ASC) undergoes oligomerization, thereby recruiting and activating caspase-1. Activated caspase-1 subsequently mediates gasdermin D cleavage and pore formation, leading to macrophage pyroptosis, while promoting the maturation and release of interleukin-1β (IL-1β) [13]. Macrophage pyroptosis not only serves as a major source of proinflammatory mediators but also exacerbates tissue injury by amplifying immune cell recruitment and disrupting pulmonary barrier integrity [14,15]. Accumulating evidence indicates that dysregulated NLRP3 inflammasome activation represents a central pathogenic mechanism in ALI and ARDS; however, the upstream signals that govern inflammasome activation and macrophage pyroptosis remain incompletely understood.

Transient receptor potential vanilloid 4 (TRPV4) is a Ca^2+^-permeable, nonselective cation channel that functions as a mechanosensor and osmosensor in the respiratory system [16,17]. Previous studies have demonstrated that TRPV4 activation contributes to pulmonary edema formation, endothelial barrier disruption, and exaggerated inflammatory responses in experimental models of lung injury [18]. Beyond its established roles in pulmonary endothelial and epithelial cells, emerging evidence suggests that TRPV4 is also expressed in macrophages and participates in innate immune regulation [19]. TRPV4-mediated Ca^2+^ influx has been reported to modulate macrophage activation, cytokine production, and responses to mechanical and osmotic stimuli in diverse inflammatory contexts [20]. During ALI, pulmonary macrophages are exposed to dynamic mechanical forces and inflammatory cues, conditions under which TRPV4 signaling is likely to be engaged. However, the role of macrophage TRPV4 in regulating inflammasome activation, pyroptotic cell death, and inflammatory amplification in ALI has not been systematically investigated.

Given the central role of macrophages in driving pulmonary inflammation and the emerging evidence implicating TRPV4 in the regulation of inflammatory signaling and cell death, we hypothesized that TRPV4 activation in macrophages functions as an upstream regulator of inflammasome-dependent pyroptosis, thereby amplifying lung inflammation and tissue injury during ALI. To test this hypothesis, we employed transcriptomic profiling in combination with genetic and pharmacological approaches in both *in vivo* and *in vitro* models to delineate the role of TRPV4 in macrophage inflammatory signaling under injurious conditions. This study aims to provide mechanistic insight into macrophage-intrinsic TRPV4 signaling in ALI and to evaluate TRPV4 as a potential therapeutic target for the treatment of ALI/ARDS.

## 2 Materials and methods

### 2.1 Mice

Male C57BL/6 mice (7-8 weeks old, 20-25 g) were purchased from Vital River Laboratory Animal Technology Co., Ltd. (Beijing, China). TRPV4^flox/flox^ and *Lyz2-Cre* mice were obtained from Cyagen Biosciences Co., Ltd. (Suzhou, China). The primers used for genotyping are listed in Table 1. All animals were housed under specific pathogen-free conditions in individually ventilated cages, with ad libitum access to sterilized water and a standard laboratory diet. The animal facility was maintained on a 12-h light/dark cycle (lights on at 08:00). All experimental procedures were reviewed and approved by the Institutional Animal Care and Use Committee of Beijing Anzhen Hospital, Capital Medical University, and were conducted in accordance with relevant institutional and national guidelines for the care and use of laboratory animals (approval no. AZ2025LA003). Animal care personnel were aware of group allocation, whereas investigators responsible for data acquisition and analysis were blinded to the treatment groups.

**Table 1:** Primer sequences for genotyping.

Primer	Forward 5′–3′	Reverse 5′–3′
TRPV4^flox/flox^	CACAGCCCTTTAAAGATTCCCTCT	ACCATGAGAGTTGTATGCAGAGAT
*Lyz2-Cre*	CTTGGGCTGCCAGAATTTCTC	CCCAGAAATGCCAGATTACG
TRPV4^flox/flox;*Lyz2*-Cre^	ATCTCAGCATGGTTAGTGTTTAGC	GTGGATTCGGACCAGTCTGA

### 2.2 Animal experiments

The animal experiments were conducted in three phases. Part I: An ALI model was established to evaluate TRPV4 expression in pulmonary macrophages. C57BL/6 mice were randomly assigned to a control (Con) or LPS group and treated with phosphate-buffered saline (PBS) or lipopolysaccharide (LPS), respectively. Lung tissues and bronchoalveolar lavage fluid (BALF) were collected 6 h later for transcriptomic sequencing, as well as histological and molecular/protein analyses. Part II: To elucidate the role of TRPV4 in ALI-associated pulmonary inflammation, an ALI model was established in myeloid cell-specific TRPV4 conditional knockout mice. TRPV4^flox/flox^ and TRPV4^CKO^ mice were randomly treated with PBS or LPS, and lung tissues and BALF were harvested 6 h later for histological and molecular/protein analyses. Part III: A pharmacological blockade strategy was employed to assess the therapeutic potential of TRPV4 inhibition. C57BL/6 mice were randomly divided into Con + Vehicle, LPS + Vehicle, and LPS + HC-067047 groups. Mice in the LPS + HC-067047 group received intraperitoneal HC-067047 (10 mg/kg, MedChemExpress, HY-100208, USA) 1 h before modeling, whereas the other groups received an equal volume of vehicle, followed by intratracheal instillation of LPS or PBS. Lung tissues and BALF were collected 6 h later for subsequent analyses. In all experiments, mice were deeply anesthetized by intraperitoneal injection of an overdose of pentobarbital sodium in accordance with institutional animal care guidelines. All samples were collected immediately for downstream processing.

### 2.3 LPS-induced ALI model

An ALI model was established via intratracheal instillation of LPS [21,22]. Mice were first anesthetized with 3% isoflurane until complete muscle relaxation and absence of limb withdrawal reflex were observed, indicating adequate anesthesia. A 20-gauge intravenous catheter was then inserted orally into the trachea and connected to a small animal ventilator. Correct placement was confirmed by observing bilateral thoracic expansion during ventilation. LPS (5 mg/kg, Sigma, E. Coli O111:B4, LPS25, USA) or PBS was then administered intratracheally through the catheter. To ensure even distribution of the instilled fluid within the lungs, mice were maintained in an upright position for 2 min. The catheter was subsequently removed, and mice were placed in a warm environment for recovery, with close monitoring of respiratory status during the awakening period.

### 2.4 RNA sequencing (RNA-seq)

Lung tissues were harvested from mice treated with PBS or LPS and submitted to BGI Genomics (Guangzhou, China) for transcriptome sequencing. Total RNA was extracted using TRIzol reagent (Invitrogen, 15596026CN) according to the manufacturer's instructions. RNA quantity and purity were assessed by spectrophotometry, and RNA integrity was evaluated by agarose gel electrophoresis based on the 18S/28S rRNA ratio. RNA sequencing libraries were constructed and sequenced on the BGISEQ-500 platform (BGI Genomics). Raw sequencing reads were quality-filtered to remove adaptor sequences and low-quality reads. Clean reads were aligned to the reference genome using Bowtie2, and gene expression levels were quantified using RNA-Seq by Expectation-Maximization (RSEM). Differentially expressed genes (DEGs) between groups were identified using DEGseq, with genes meeting the criteria of |log_2_ fold change| > 0.5 and false discovery rate (FDR, q-value) < 0.05 considered significantly differentially expressed. Gene Ontology (GO) and Kyoto Encyclopedia of Genes and Genomes (KEGG) pathway enrichment analyses were performed for DEGs using the hypergeometric test (Phyper), with q-value ≤0.05 defined as statistically significant enrichment. In addition, functional annotation and enrichment analyses were conducted using the DAVID online tool. Gene Set Enrichment Analysis (GSEA) was performed to identify coordinated changes in predefined gene sets across the transcriptome.

To further evaluate the relationship between TRPV4 expression and inflammasome-related pathway activity, Gene Set Variation Analysis (GSVA) was applied to calculate pathway activity scores, which were subsequently analyzed in relation to TRPV4 expression levels in the lungs of control and ALI mice.

### 2.5 RNA-seq analysis from public datasets

RNA-seq data from lung tissues of mice with ALI were downloaded from the Gene Expression Omnibus database (accession number GSE263867). Raw count matrices were imported into R for downstream analysis. Differential gene expression analysis between ALI and control groups was performed using the DESeq2 package. Genes with an adjusted p value < 0.05 and |log2 fold change| ≥ 1 were defined as differentially expressed genes (DEGs). Volcano plots and heatmaps were generated to visualize gene expression changes. Gene Ontology (GO) enrichment analysis and KEGG pathway analysis of DEGs were conducted using R (v4.5.2) to identify significantly enriched biological processes and pathways, and the results were visualized as bubble plots.

### 2.6 Cell isolation, differentiation and treatment

Mouse bone marrow-derived macrophages **(**BMDMs) were isolated following previously described methods. Briefly, TRPV4^CKO^ and TRPV4^flox/flox^ mice aged between 6 and 8 weeks were sacrificed in a CO_2_ chamber, and the tibia and femora were isolated. Bone marrow cells were flushed out from the bone, resuspended and cultured in RPMI 1640 medium (Gibco, 11875-093, USA) supplemented with 1% penicillin-streptomycin solution (Gibco, 15640055, USA), 20 ng/ml M-CSF (MedChemExpress, HY-P7085, USA), and 10% fetal bovine serum (Sigma, F8318, USA) at 37 °C in a 5% CO_2_ incubator. Fresh complete medium was replenished on days 3 and 6. After 7 days of differentiation, adherent BMDMs were harvested and plated at a density of 1 × 10^6^ cells/mL for subsequent experiments. The murine alveolar macrophage cell line MH-S was obtained from the American Type Culture Collection and cultured in RPMI 1640 medium supplemented with 10% FBS and 1% penicillin–streptomycin solution. For pharmacological treatments, MH-S cells were pretreated with intracellular calcium chelator BAPTA-AM (BAPTA; 10 μM, 30 min, MedChemExpress, HY-100545, USA), the TRPV4 agonist GSK1016790A (GSK; 300 nM, 30 min, MedChemExpress, HY-19708), the mitochondrial reactive oxygen species (mtROS) scavenger mito-TEMPO (TEMPO; 1 μM, 30 min, MedChemExpress, HY-112879, USA), or the TRPV4 antagonist HC-067047 (1 μM, 30 min), followed by stimulation with LPS (1 μg/mL) for 6 h and adenosine triphosphate (ATP; 5 mM, MedChemExpress, HY-B2176, USA) for 30 min.

### 2.7 Flow cytometry and cell sorting

BMDMs were harvested and washed twice with Hank's balanced salt solution (HBSS, Thermo, 14175095, USA). Cells were incubated with anti-mouse CD16/32 (Elabscience, E-AB-F0997A, China) for 15 min at room temperature to block Fc receptors. Subsequently, cells were stained with fluorochrome-conjugated antibodies against 7-AAD (Elabscience, E-CK-A162, China), BV450 Anti-Mouse CD45 antibody (Elabscience, E-AB-F1136Q, China), PE Anti-Mouse F4/80 antibody (Elabscience, E-AB-F0995D, China), FITC Anti-Mouse Ly6G antibody (Elabscience, E-AB-F1108C, China), and APC Anti-Mouse/Human CD11b antibody (Elabscience, E-AB-F1081E, China) on ice for 30 min in the dark. After two additional washes, samples were analyzed using a BD FACS AriaIIIu flow cytometer (BD Biosciences, USA), and data were processed with FlowJo software (v10.0; FlowJo, LLC). BMDMs were defined as CD45^+^ Ly6G^-^ F4/80^+^ CD11b^+^ cells, and this gating strategy was used to assess the purity of the isolated macrophage population.

### 2.8 Calcium imaging

BMDMs were seeded on glass-bottom dishes and loaded with the calcium-sensitive fluorescent dye Fluo-4 AM (5 μM, Thermo, F10489, USA) in HBSS at 37 °C for 30 min in the dark, followed by an additional 30 min incubation at room temperature to allow complete de-esterification. After washing to remove excess dye, cells were maintained in fresh HBSS. The intracellular Ca^2+^ dynamics were recorded using a Leica STELLARIS fluorescence microscope (Leica, Germany) with an excitation wavelength of 488 nm and an emission range of 505–530 nm. Fluorescence signals were captured at 2 s intervals over a 3 min period. Then cells were stimulated with GSK (300 nM), and fluorescence signals were recorded for an additional 180 s. The fluorescence intensity data were analyzed with the Leica Application Suite X (Leica, Germany) software. Fluo-4 AM is a single wavelength indicator whose fluorescence varies monotonically with Ca^2+^ but does not allow direct calculation of absolute concentrations without in situ calibration. Therefore, all data are expressed as the fractional fluorescence change relative to baseline (ΔF/F_0_ = (F_t_-F_0_)/F_0_), where F_0_ denotes the mean fluorescence intensity during the baseline recording period [23]. This metric reflects the magnitude of Ca^2+^ transients independent of dye loading variability.

### 2.9 Small interfering RNA (siRNA) transfection

siRNAs for the mouse TRPV4 gene were obtained from GeneChem (Shanghai, China). Macrophages were plated in a six-well plate at a density of 2.0 × 10^5^ cells per well and cultured for 24 h to 60–70% confluence. The cells were transfected with 50 nM negative control or siRNA duplexes using Lipofectamine RNAiMAX (Thermo, 13778150, USA) for 24 h. Subsequently, the transfection efficiency of TRPV4 was evaluated by real-time quantitative reverse transcription polymerase chain reaction (RT-qPCR). The siRNA targeting sequences used in this study are as follows:

Sense: 5′-GGAGAAAGGUCGUGGAGAA-3′

Antisense: 5′-UUCUCCACGACCUUUCUCC-3′

### 2.10 BALF collection and analysis

Following anesthesia, a 20-gauge catheter was carefully inserted into the trachea. The lungs were flushed three times with 1 mL of pre-cooled, sterile PBS using a 1 mL syringe to collect BALF [21,24]. The recovered fluid was immediately centrifuged at 1500 rpm for 15 min at 4 °C. The supernatant was aliquoted for subsequent measurement of total protein levels and inflammatory mediators. Protein concentrations were assessed using a bicinchoninic acid assay (BCA) kit (Thermo, 23227, USA) according to the manufacturer's protocol.

### 2.11 Diff-Quik staining

Cells in BALF were cytospun onto glass slides, air-dried, and stained using a Diff-Quik staining kit (Solarbio, G1540, China) according to the manufacturer's instructions. Briefly, slides were sequentially immersed in fixative solution, eosinophilic dye, and basophilic dye, followed by gentle rinsing with distilled water. After air-drying, the stained cells were examined under a light microscope for morphological evaluation and differential cell counting, with particular emphasis on the identification and quantification of polymorphonuclear neutrophils (PMNs) [21].

### 2.12 Hematoxylin eosin (HE) staining and lung injury scoring

Lung tissues were fixed in 4% paraformaldehyde, dehydrated through a graded ethanol series, embedded in paraffin, and sectioned into 5 μm slices using a microtome. Sections were deparaffinized with xylene and rehydrated through descending concentrations of ethanol. Standard HE staining was then performed. After staining, sections were mounted with neutral balsam and examined under a light microscope. Histopathological assessment of lung injury was conducted using a semiquantitative scoring system as previously described [25]. Four independent criteria were evaluated: (1) alveolar and/or interstitial edema, (2) hemorrhage, (3) neutrophil infiltration or aggregation in the alveolar space or vessel walls, and (4) thickening of the alveolar septa and/or formation of hyaline membranes. Each parameter was scored on a scale of 0 to 4 based on the severity and extent of injury: 0 = no injury; 1 = mild, localized involvement (<25% of the field); 2 = moderate (25-50%); 3 = marked (50-75%); and 4 = severe, widespread damage (>75%). The total lung injury score was calculated by summing the individual scores, yielding a maximum of 16 points. Scoring was independently performed by two blinded investigators, and the final score was obtained by averaging the two results when discrepancies occurred.

### 2.13 Lung wet-to-dry weight (W/D) ratio

The right upper lobe of the lung was excised and immediately weighed to obtain the wet weight. The tissue was then placed in a drying oven at 65 °C for 72 h until a constant weight was achieved, representing the dry weight. The W/D ratio was calculated by dividing the wet weight by the corresponding dry weight, serving as an indicator of pulmonary edema severity [15,21,22].

### 2.14 Assessment of pulmonary vascular permeability

To evaluate pulmonary vascular leakage, Evans blue dye (EBD, 30 mg/kg, Sigma, E2129, USA) was administered via the tail vein 1 h prior to lung tissue collection [21,26]. Mice were perfused with cold PBS containing 0.6 mmol/L EDTA to remove intravascular dye. The lungs were then blotted dry, weighed, and snap-frozen in liquid nitrogen. For dye extraction, 100 mg of frozen lung tissue was homogenized in 0.5 mL PBS, followed by the addition of 1.0 mL formamide (MedChemExpress, HY-Y0842, USA). Samples were incubated at 60 °C for 18 h and then centrifuged at 10,000 × g for 20 min. The absorbance of the supernatant was measured at 620 nm and 740 nm using a spectrophotometer. To correct for interference from heme pigments, the following formula was applied: Corrected A620 = Measured A620- (1.1649 × A740 + 0.004) [21]. The Evans blue index was calculated as the amount of dye extracted per gram of lung tissue, serving as a quantitative indicator of vascular permeability.

### 2.15 Enzyme-linked immunosorbent assay (ELISA)

The concentrations of the proinflammatory cytokines tumor necrosis factor-α (TNF-α) and IL-1β in the cell culture supernatant and BALF were quantified using the mouse TNF-α ELISA kit (Elabscience, E-EL-M3063, China) and the mouse IL-1β ELISA kit (Elabscience, E-EL-M0037, China), respectively, following the manufacturer's instructions.

### 2.16 Western blot analysis

Lung tissues or cultured cells were harvested, lysed, and protein concentrations were determined using a BCA protein assay kit [27] (Thermo, 23227, USA). Equal amounts of protein were separated by 8-12% SDS-PAGE and subsequently transferred onto 0.22 μm nitrocellulose membranes (Millipore, 0000445441, USA). After blocking with 5% skim milk, membranes were incubated with primary antibodies overnight at 4 °C. The primary antibodies used were as follows: TRPV4 (1:1000, Abcam, ab39260, UK), NLRP3 (1:1000, Abcam, ab263899, UK), caspase-1 (1:1000, Abcam, ab179515, UK), IL-1β (1:1000, Abcam, ab234437, UK), apoptosis-associated speck-like protein containing a CARD (ASC; 1:1000, Abcam, ab309497, UK), N-terminal gasdermin D (N-GSDMD; 1:1000, Abcam, ab209845, UK), hexokinase 2 (HK2; 1:1000, Abcam, ab209847, UK), pyruvate dehydrogenase kinase 1 (PDK1; 1:1000, Abcam, ab110025, UK), lactate dehydrogenase A (LDHA; 1:2000, Abcam, ab135396, UK), NADH: ubiquinone oxidoreductase subunit B8 (NDUFB8; 1:1000, Proteintech, 14794-1-AP, China), succinate dehydrogenase complex iron–sulfur subunit B (SDHB; 1:1000, Proteintech, 10620-1-AP, China), ubiquinol-cytochrome c reductase core protein 2 (UQCR2; 1:1000, Proteintech, 14742-1-AP, China), mitochondrially encoded cytochrome *c* oxidase I (MTCO1; 1:1000, Abcam, ab14705, UK), ATP synthase F1 subunit alpha (ATP5A1; 1:1000, Proteintech, 14676-1-AP, China), and β-actin (1:2500, Abcam, ab8226, UK). After washing, membranes were incubated with appropriate horseradish peroxidase-conjugated secondary antibodies (EarthOx, E030120, USA) for 1 h at room temperature. Protein bands were visualized using an Amersham Imager 600 (GE Healthcare, USA), and quantified using ImageJ software (Scion Co., Walkersville, USA). Target protein expression was normalized to β-actin as the loading control, and data were further normalized to the corresponding control group and expressed as fold change relative to control.

### 2.17 Immunohistochemistry (IHC)

Paraffin-embedded lung tissue sections were deparaffinized, rehydrated, and subjected to antigen retrieval. Endogenous peroxidase activity was quenched with 3% H_2_O_2_, followed by blocking with 5% goat serum. The sections were incubated with TRPV4 antibody (1:100) overnight at 4 °C, followed by incubation with a secondary antibody and visualization using 3,3′-Diaminobenzidine. The nuclei were counterstained with hematoxylin, and the slides were dehydrated and mounted. Images were captured under a light microscope. TRPV4 expression was semi-quantitatively evaluated based on staining intensity and positive area in randomly selected fields, and normalized to the corresponding control group.

### 2.18 Lung tissue immunofluorescence

Immunofluorescence staining of frozen lung tissue sections was performed as previously described [27]. The sections were incubated overnight at 4 °C with F4/80 antibody (1:400, Abcam, ab6640, UK), N-GSDMD antibody (1:200, Immunoway, YM4266, USA), or ASC antibody (1:200, Immunoway, YM8352, USA). Subsequently, Alexa Fluor 488-conjugated secondary antibody (1:1000, Abcam, ab150077, UK) or Alexa Fluor 594-conjugated secondary antibody (1:1000, Abcam, ab150080, UK) were applied for fluorescence detection. For quantitative analysis, six random non-overlapping fields per section were selected under identical imaging settings across all groups. Fluorescence intensity was quantified using ImageJ software. Mean fluorescence intensity (MFI) was calculated and normalized to control samples. Data were obtained from six independent experiments and used for statistical analysis.

### 2.19 Tyramide signal amplification (TSA) -based immunofluorescence staining

Paraffin-embedded lung tissue sections were deparaffinized, rehydrated, and subjected to antigen retrieval in citrate buffer (pH 6.0). Endogenous peroxidase activity was quenched with 3% hydrogen peroxide, followed by blocking with 5% BSA for 30 min at room temperature. Sections were incubated overnight at 4 °C with primary antibodies against TRPV4 (1:100), CD31 (1:200, Proteintech, 28083-1-AP, China), or SP-C (1:200, Proteintech, 107740-1-AP, China). After washing, HRP-conjugated secondary antibodies were applied, followed by TSA kit according to the manufacturer's instructions (Abcam, ab312827, UK). Sequential staining was performed for multiplex detection when required [28]. Nuclei were counterstained with 4′,6-diamidino-2-phenylindole (DAPI, Beyotime, C1002, China). Slides were mounted with antifade medium and imaged using fluorescence microscopy under identical acquisition settings across groups. Co-localization analysis was performed using ImageJ software.

### 2.20 Transmission electron microscopy (TEM) analysis

Collected lung tissues and macrophages primed with LPS plus ATP were immediately immersed in pre-chilled 2.5% glutaraldehyde for 24 h, post-fixed in 1% osmium tetroxide, dehydrated through a graded ethanol series, and infiltrated with epoxy resin followed by polymerization at 60 °C to obtain hardened blocks. Ultrathin sections (50 nm) were prepared using an ultramicrotome, mounted onto copper grids, and sequentially contrasted with uranyl acetate and lead citrate. Samples were examined under a transmission electron microscope at 8000-12000× to visualize cellular architecture and membrane ultrastructure.

Pyroptotic macrophages were identified based on characteristic features, including pronounced cell swelling, cytoplasmic vacuolization, loss of plasma-membrane integrity with pore-like discontinuities, chromatin decondensation or marginalization, and organelle degeneration, and representative images were documented. For mitochondrial morphometric analysis, at least six randomly selected electron micrographs from each sample were analyzed using ImageJ software. The following mitochondrial parameters were quantified: surface area (μm^2^), perimeter (μm), Feret's diameter (defined as the longest distance between any two points within a mitochondrion), and aspect ratio (defined as the ratio of the major axis to the minor axis, reflecting mitochondrial elongation) [29,30].

### 2.21 RNA isolation and quantification

Total RNA was extracted from mouse lung tissue or macrophages using TRIzol following the manufacturer's protocol. RNA quality and concentration were measured by NanoDrop (Thermo, USA). cDNA was synthesized with the PrimeScript™ RT kit (Accurate biology, AG11728, China). Quantitative PCR was performed using SYBR Green Master Mix (Thermo, A25742, USA) on a StepOnePlus Real-Time PCR System (Applied Biosystems, USA). Gene expression was calculated by the 2^−ΔΔCt^ method [31], with β-actin as the internal reference. The primers are listed in Table 2.

**Table 2:** Primer sequences for RT-qPCR.

Primer	Forward 5′–3′	Reverse 5′–3′
IL-1β	GCAGAGCACAAGCCTGTCTTCC	ACCTGTCTTGGCCGAGGACTAAG
TNF α	ATGTCTCAGCCTCTTCTCATTC	GCTTGTCACTCGAATTTTGAGA
ICAM-1	GTGATGCTCAGGTATCCATCCA	CACAGTTCTCAAAGCACAGCG
VCAM-1	CGCCAGGAATCTGTAACAAAACA	CAGTACACGAGCCTGTGCTT
MCP-1	TTAAAAACCTGGATCGGAACCAA	GCATTAGCTTCAGATTTACGGGT
β-actin	GGCTGTATTCCCCTCCATCG	CCAGTTGGTAACAATGCCATGT

### 2.22 Measurement of intracellular Ca^2+^

Macrophages were incubated with the Ca^2+^-sensitive fluorescent indicator Fluo-4 AM (5 μM) at 37 °C for 30 min to achieve dye loading. Cells were then washed three times with buffer to remove unbound dye and further incubated at 37 °C for 30 min to allow complete intracellular de-esterification of AM esters. Subsequently, nuclei were stained with Hoechst 33342 (10 μg/mL, Solarbio, C0031, China) for 10 min, followed by three additional washes to eliminate excess dye. The culture medium was then replaced with serum-free medium for intracellular Ca^2+^ measurement. Fluorescence signals reflecting intracellular Ca^2+^ concentration were acquired using a Leica SP8 fluorescence microscope (Leica, Germany).

### 2.23 Flow cytometric analysis of cell death profile

The overall cell death profile was first characterized using an Annexin V-FITC/propidium iodide (PI) staining kit (Elabscience, E-CK-A211B, China) according to the manufacturer's instructions. Briefly, MH-S cells (1.0 × 10^6^) were harvested using an EDTA-free dissociation solution, centrifuged, and washed twice with ice-cold PBS. Cells were resuspended in 100 μL Annexin binding buffer and incubated with 5 μL Annexin V-FITC and 5 μL PI for 20 min at room temperature in the dark. After the addition of 400 μL binding buffer, samples were analyzed using a Thermo Attune CytPix flow cytometer (Thermo Fisher Scientific, USA). Data were processed with FlowJo software (version 10.10, BD Biosciences, USA). Cell populations were classified as viable (Annexin V^−^/PI^−^), Annexin V-positive (Annexin V^+^/PI^−^ and Annexin V^+^/PI^+^), and PI-positive cells, and the cell death profile was quantified based on the distribution of these populations.

### 2.24 Flow cytometric analysis of pyroptosis-associated cell death

Pyroptosis-associated cell death was subsequently evaluated using a Hoechst/PI staining kit (Beyotime, C1056, China). Briefly, Cells (1.0 × 10^6^) were trypsinized, centrifuged, and resuspended in 500 μL of staining solution. Subsequently, 5 μL of Hoechst and PI staining reagents were added, gently mixed, and incubated at 4 °C in the dark for 20 min. The percentage of pyroptotic cells in each group was then determined using a flow cytometer (Beckman Coulter, USA). PI-positive cells were considered to represent membrane-compromised cells, indicative of pyroptosis-associated cell death when interpreted in conjunction with molecular markers such as N-GSDMD and Cl-caspase-1 activation.

### 2.25 Mitochondrial membrane potential (ΔΨm)

Macrophages were seeded in confocal dishes at a density of 1.0 × 10^5^ cells per dish. Mitochondrial membrane potential was evaluated using a JC-1 detection kit (MedChemExpress, HY-15534, USA). After incubation with 2 μM JC-1 dye at 37 °C for 30 min [21], cells were washed with HBSS, and the red/green fluorescence ratio was assessed under a fluorescence microscope (Leica SP8 STED, Germany). ΔΨm was quantified by calculating the ratio of red (J-aggregates) to green (monomers) fluorescence using ImageJ software. Six randomly selected non-overlapping fields per sample were analyzed under identical imaging settings across all groups. Data were obtained from six independent experiments for statistical analysis.

### 2.26 MitoTracker staining

Cells were plated in confocal dishes at 1.0 × 10^5^ cells per dish. Mitochondria were labeled with MitoTracker Red CMXRos (5 μM, MedChemExpress, HY-D1116, USA) at 37 °C for 30 min, followed by HBSS washing and Hoechst nuclear staining for 5 min [32]. Fluorescent signals were then imaged using a fluorescence microscope (Leica SP8 STED, Germany). Mitochondrial fluorescence intensity was quantified using ImageJ software. Six randomly selected non-overlapping fields per sample were analyzed under identical acquisition settings, and signal intensity was normalized to control. Data were derived from six independent experiments and used for statistical analysis.

### 2.27 mtROS detection

Macrophages were plated in confocal dishes at a density of 1.0 × 10^5^ cells per dish. mtROS levels were assessed using MitoSOX Red (MedChemExpress, HY-D1055, USA) via confocal imaging and flow cytometry. For confocal analysis, cells were incubated with 8 μM of the dye at 37 °C for 30 min, washed with HBSS, and nuclei were counterstained with Hoechst for 5 min [33]. Fluorescence images were acquired using a confocal laser scanning microscope (Leica SP8 STED, Germany) under identical exposure settings across all groups. Quantification was performed using ImageJ by measuring the MFI of six randomly selected, non-overlapping fields per sample, with values normalized to the control group.

For quantitative analysis, mtROS levels were further measured by flow cytometry. After MitoSOX staining under the same conditions, cells were harvested, washed, and resuspended in HBSS. Fluorescence signals were analyzed using a flow cytometer (Beckman Coulter, USA), and data were further processed using FlowJo software (version 10.10, BD Biosciences, USA). The MFI of MitoSOX fluorescence was quantified and normalized to the control group. All experiments were performed with consistent staining conditions to minimize dye-loading variability.

### 2.28 Measurement of cellular ATP levels

Intracellular ATP content was determined using an ATP assay kit (Beyotime, S0027, China) following the manufacturer's instructions [34]. ATP levels were measured with a microplate luminometer (BioTek, USA) and normalized to the values of the control group.

### 2.29 Measurement of mitochondrial oxygen consumption rate (OCR)

OCR was assessed using the Seahorse XFe24 Analyzer (Agilent Technologies, USA) in combination with the Seahorse XF Cell Mito Stress Test Kit (Agilent, 103015-100, USA), following the manufacturer's guidelines. Briefly, cells were seeded at 2.0 × 10^4^ cells per well in Seahorse 24-well plates pre-coated with a cell-adhesion reagent and allowed to adhere overnight. Prior to the assay, cells were washed, and the medium was replaced with Seahorse XF RPMI medium supplemented with 20 mM glucose, 2 mM l-glutamine, and 1 mM sodium pyruvate [9,35], and equilibrated in a non-CO_2_ incubator for 60 min. OCR was measured under basal conditions and following sequential injections of oligomycin, carbonyl cyanide-p-trifluoromethoxyphenylhydrazone (FCCP), and rotenone/antimycin A to assess ATP-linked respiration, maximal respiratory capacity, and non-mitochondrial respiration, respectively. Basal respiration was calculated by subtracting non-mitochondrial respiration (rotenone/antimycin A-insensitive OCR) from basal OCR. ATP-linked respiration was calculated as the difference between basal OCR and oligomycin-inhibited OCR. Maximal respiration was determined following FCCP stimulation, and non-mitochondrial respiration was defined as the residual OCR after rotenone/antimycin A treatment. OCR values were normalized to the initial seeding density (2.0 × 10^4^ cells per well) and expressed as per-cell OCR. All experiments were performed with technical replicates and averaged across independent biological repeats.

### 2.30 N-GSDMD immunofluorescence staining

Cells on coverslips were fixed with 4% paraformaldehyde for 15 min, permeabilized with 0.3% Triton X-100 for 10 min, and blocked with 5% normal goat serum for 1 h at room temperature. Cells were then incubated with anti-N-GSDMD antibody (1:200) overnight at 4 °C, followed by Alexa Fluor 594-conjugated secondary antibody for 1 h in the dark. Nuclei were counterstained with DAPI, and coverslips were mounted with anti-fade medium for fluorescence imaging. For quantification, six non-overlapping fields per sample were randomly selected under identical exposure settings across all groups, and the MFI of N-GSDMD was analyzed using ImageJ software and normalized to the control group. Data were derived from six independent experiments.

### 2.31 Statistical analysis

Data were presented as the mean ± standard deviation and analyzed using GraphPad Prism 10 statistical software (San Diego, CA). Two groups were compared using the Student's t-test, and multiple groups were compared using one-way analysis of variance (One-way ANOVA), followed by Tukey's post-hoc test for pairwise comparisons. *P* less than 0.05 was considered statistically significant.

## 3 Results

### 3.1 Upregulation of TRPV4 in macrophage is associated with excessive inflammation in ALI lungs

**Fig. 1:**
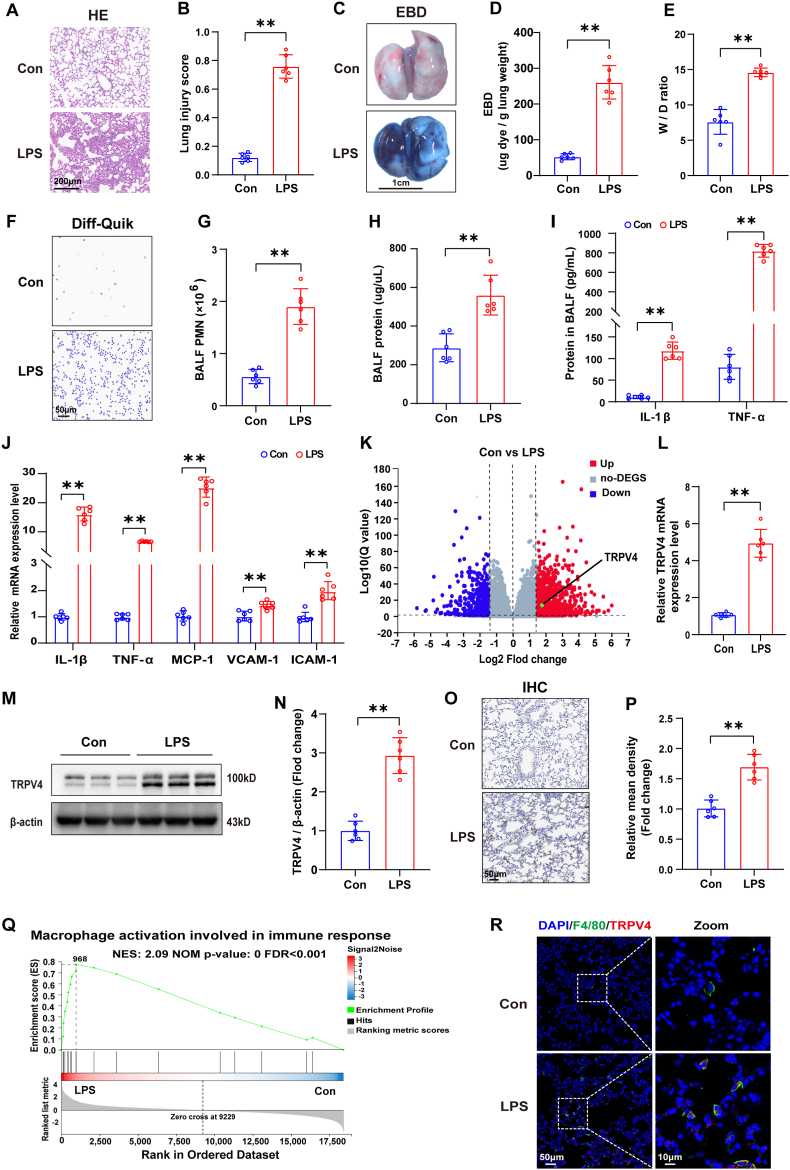
TRPV4 expression is upregulated in the macrophage of ALI mice A, Representative macroscopic images of lung sections stained with hematoxylin and eosin (HE) from mice treated with PBS or LPS. scale bar: 200 μm. B, Quantification of pathological lung injury scores based on HE staining. n = 6. C, Representative images of lung stained with evans blue dye (EBD) from mice treated with PBS or LPS. scale bar:1 cm. D, Quantitative analysis of Evans Blue-labeled albumin in mice treated with PBS or LPS. n = 6. E, Lung wet/dry (W/D) ratio in mice treated with PBS or LPS, n = 6. F, Representative images of bronchoalveolar lavage fluid (BALF) cytospin preparations from mice treated with PBS or LPS, stained with Diff-Quik. scale bar: 50 μm. G, Polymorphonuclear neutrophils (PMNs) count in BALF from mice treated with PBS or LPS, determined by Diff-Quik staining. n = 6. H, Protein concentrations in BALF from mice treated with PBS or LPS. n = 6. I, Levels of IL-1β and TNF-α in BALF from mice treated with PBS or LPS. n = 6. J, Expression of proinflammatory cytokine (IL-1β, TNF-α, MCP-1, VCAM-1, ICAM-1) mRNA in the lungs of mice treated with PBS or LPS. n = 6. K, Enrichment of differentially expressed genes (DEGs) in lungs from ALI versus normal. L, Expression of TRPV4 mRNA in lungs from mice treated with PBS or LPS. n = 6. M − N, Western blot and quantification of TRPV4 protein levels in lungs from mice treated with PBS or LPS. n = 6. O–P, Representative macroscopic images and quantification of immunohistochemical (IHC) staining of TRPV4 in the lung sections from mice treated with PBS or LPS. n = 6. Q, Gene set enrichment analysis (GSEA) of DEGs from LPS-treated and control lungs. R, Immunofluorescence staining of F4/80 (green) and TRPV4 (red) in lung sections from mice treated with PBS or LPS. Nuclei: DAPI. Scale bar: 50 μm and 10 μm. All data are mean ± SD. Statistical significance was determined by Student's t-test. ∗*P* < 0.05, ∗∗*P* < 0.01 vs. Con group.

Given the pivotal role of excessive inflammation in the pathogenesis of ALI, we first established a murine ALI model by intratracheal administration of LPS (5 mg/kg); then, after 6 h, relevant inflammatory parameters were examined. As shown in Fig. 1, intratracheal administration of LPS induced severe lung injury, characterized by marked thickening of the alveolar septa, extensive disruption of alveolar architecture, and pronounced inflammatory cell infiltration (Fig. 1A), accompanied by a significant increase in lung injury scores (Fig. 1B). Consistently, LPS challenge resulted in marked alveolar–capillary barrier dysfunction, as evidenced by enhanced EBD extravasation in lung tissues (Fig. 1C–D) and a significantly elevated lung W/D ratio (Fig. 1E). Analysis of BALF further demonstrated a robust inflammatory response, with significantly increased PMNs counts (Fig. 1F and G), elevated total protein levels (Fig. 1H), and markedly increased concentrations of the pro-inflammatory cytokines IL-1β and TNF-α (Fig. 1I). In parallel, mRNA expression levels of IL-1β, TNF-α, intercellular adhesion molecule-1 (ICAM-1), monocyte chemoattractant protein-1 (MCP-1), and vascular cell adhesion molecule-1 (VCAM-1) were significantly upregulated in lung tissues following LPS exposure (Fig. 1J).

To identify potential molecular targets involved in the regulation of excessive pulmonary inflammation, bulk RNA sequencing was performed on lung tissues. Differential gene expression analysis revealed a significant upregulation of TRPV4 in the LPS-treated group (Fig. 1K). Reanalysis of an independent RNA sequencing dataset (GSE263867) consistently confirmed a marked upregulation of TRPV4 expression in the lungs of mice with LPS-induced ALI (Supplementary Fig. S1A). These observations were further corroborated at both the mRNA and protein levels by RT-qPCR (Fig. 1L), Western blot analysis (Fig. 1M and N), and immunohistochemical staining (Fig. 1O and P), all of which demonstrated increased TRPV4 expression in the lungs of LPS-challenged mice. GSEA revealed that immune response-related pathways, particularly those associated with macrophage activation, were significantly enriched in the LPS group (Fig. 1Q). Consistently, GO analysis of the GSE263867 dataset also highlighted macrophage activation as a prominent biological process in ALI lungs (Supplementary Fig. S1B). To further determine the cellular distribution of TRPV4 in ALI lungs, immunofluorescence staining was performed. TRPV4 showed minimal colocalization with the endothelial cell marker CD31 and the alveolar epithelial cell marker SP-C (Supplementary Fig. S1C), whereas prominent colocalization of TRPV4 with macrophages was observed in lung tissues from LPS-treated mice (Fig. 1R and Supplementary Fig. S1D). Collectively, these findings indicate that TRPV4 is significantly upregulated in macrophages during LPS-induced lung inflammation.

### 3.2 Myeloid-specific TRPV4 deficiency attenuates inflammation and lung injury in ALI

**Fig. 2:**
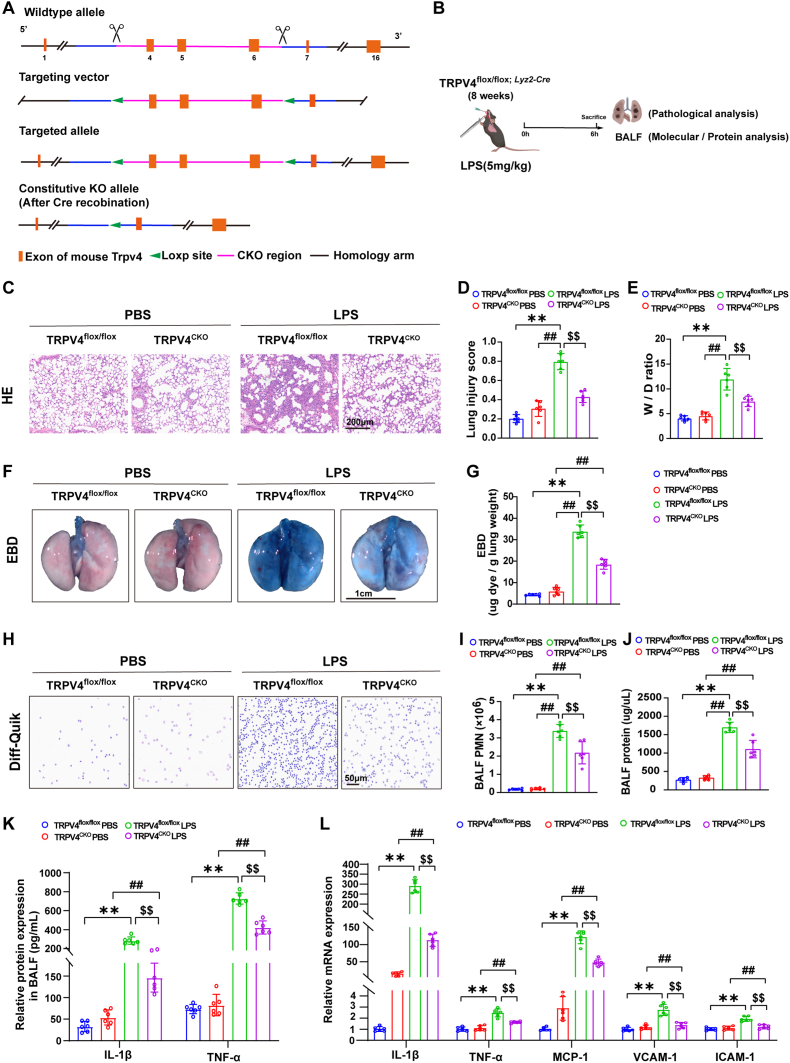
Myeloid cell-specific TRPV4 deficiency attenuates inflammation and lung injury in mice A, Strategy for generating TRPV4^CKO^ mice. Vector construction: mouse genomic fragments were assembled into a targeting vector together with recombination sites and selection markers, as indicated on the vector map. B, Schematic of the experimental design for the LPS-induced ALI in TRPV4^flox/flox^ and TRPV4^CKO^ mice. C, Representative images of HE-stained lung sections from TRPV4^flox/flox^ and TRPV4^CKO^ mice treated with LPS or PBS. Scale bar: 200 μm. D, Quantification of pathological lung injury scores based on HE staining. n = 6. E, Lung W/D ratio in TRPV4^flox/flox^ and TRPV4^CKO^ mice treated with LPS or PBS. n = 6. F, Representative images of lung stained with EBD in TRPV4^flox/flox^ and TRPV4^CKO^ mice treated with LPS or PBS. Scale bar: 1 cm. G, Quantitative analysis of Evans Blue-labeled albumin in TRPV4^flox/flox^ and TRPV4^CKO^ mice treated with LPS or PBS. n = 6. H, Representative images of BALF cytospin preparations stained with Diff-Quik in TRPV4^flox/flox^ and TRPV4^CKO^ mice with PBS or LPS. Scale bar: 50 μm. I, PMNs count in BALF determined by Diff-Quik staining in TRPV4^flox/flox^ and TRPV4^CKO^ mice. n = 6. J, Protein concentrations in BALF from TRPV4^flox/flox^ and TRPV4^CKO^ mice treated with PBS or LPS. n = 6. K, Expression of proinflammatory cytokine (IL-1β, TNF-α, MCP-1, VCAM-1, ICAM-1) mRNA in lungs of TRPV4^flox/flox^ and TRPV4^CKO^ mice treated with PBS or LPS. n = 6. L, Levels of IL-1β and TNF-α in the BALF of TRPV4^flox/flox^ and TRPV4^CKO^ mice with PBS or LPS. n = 6. All data are mean ± SD. Statistical significance was determined by one-way ANOVA followed by Tukey's post-hoc test. ∗*P* < 0.05, ∗∗*P* < 0.01 vs. TRPV4^flox/flox^ PBS group; ^#^*P* < 0.05, ^##^*P* < 0.01 vs. TRPV4^CKO^ PBS group; ^$^*P* < 0.05, ^$$^*P* < 0.01 vs. TRPV4^flox/flox^ LPS group.

Next, to explore whether TRPV4 in macrophages contributes to inflammatory responses of ALI, myeloid-specific TRPV4 conditional knockout mice (TRPV4^CKO^) were generated. The TRPV4^CKO^ (TRPV4^flox/flox;^ ^*Lyz2-Cre*^) mice were obtained by crossing TRPV4^flox/flox^ mice with *Lyz2-Cre* mice (Fig. 2A and Supplementary Fig. S2A–G). Eight-week-old TRPV4^flox/flox^ and TRPV4^CKO^ mice were subjected to intratracheal administration of LPS (5 mg/kg) for 6 h to induce ALI. As a result, HE revealed that, compared with LPS-treated TRPV4^flox/flox^ mice, TRPV4^CKO^ mice exhibited markedly reduced alveolar septal thickening, relatively preserved alveolar architecture, and decreased inflammatory cell infiltration (Fig. 2C), accompanied by a significant reduction in lung injury scores (Fig. 2D). Consistently, lung edema was alleviated in TRPV4^CKO^ mice, as indicated by a significantly decreased lung W/D ratio (Fig. 2E). In addition, alveolar-capillary barrier integrity was markedly improved in TRPV4^CKO^ mice, as evidenced by reduced EBD extravasation in lung tissues (Fig. 2F and G).

Analysis of BALF demonstrated that TRPV4 deficiency in myeloid cells significantly attenuated inflammatory cell recruitment, with markedly reduced neutrophil counts (Fig. 2H and I), decreased total protein levels (Fig. 2J), and significantly lower concentrations of the pro-inflammatory cytokines IL-1β and TNF-α (Fig. 2K). Furthermore, mRNA expression levels of IL-1β, TNF-α, ICAM-1, MCP-1, and VCAM-1 in lung tissues were significantly downregulated in TRPV4^CKO^ mice following LPS challenge (Fig. 2L). Taken together, these results suggested that myeloid-specific TRPV4 deficiency confers significant protection against LPS-induced pulmonary inflammation and lung injury.

### 3.3 TRPV4 upregulation correlates with NLRP3 activation and macrophage pyroptosis in the inflamed ALI lung

**Fig. 3:**
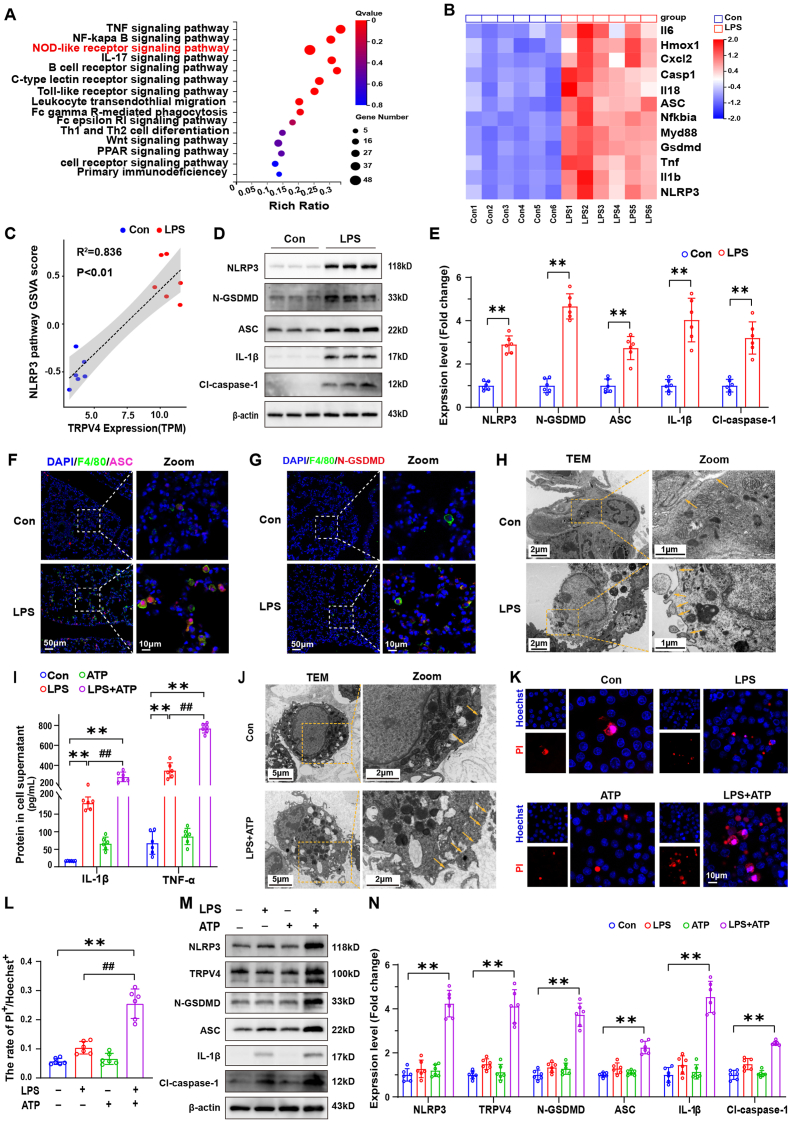
Upregulation of TRPV4 in the inflamed lung is accompanied by enhanced NLRP3 inflammasome activation and macrophage pyroptosis A, Kyoto Encyclopedia of Genes and Genomes (KEGG) pathway enrichment analysis of upregulated DEGs in lungs from Con and ALI mice. B, Heatmap of markers in the NOD-like receptor signaling pathway. Colors represent Z-scores. C, Correlation between TRPV4 expression and NLRP3 pathway activity in the lungs of control and ALI mice, assessed by Gene Set Variation Analysis (GSVA). D-E, Western blot analysis and quantification of NOD-like receptor pyrin domain-containing protein 3 (NLRP3), Cl-caspase-1, IL-1β, apoptosis-associated speck-like protein containing a CARD (ASC), and N-terminal gasdermin D (N-GSDMD) protein levels in mice treated with PBS or LPS. n = 6. F-G, Representative immunofluorescence images exhibiting co-immunostaining of ASC (red) or N-GSDMD (red) with F4/80 (green) in lung sections of mice treated with PBS or LPS. Scale bar: 50 μm and 10 μm. H, Representative transmission electron microscopy (TEM) images of macrophages in lungs treated with PBS or LPS. Scale bar: 2 μm and 1 μm. I, Levels of IL-1β and TNF-α in the supernatants of LPS + ATP-stimulated macrophages. n = 6. J, Representative TEM images showing ultrastructural mitochondrial alterations in macrophages stimulated with LPS + ATP. Scale bar: 5 μm and 2 μm. K–L, Representative immunofluorescence images and quantification of PI (red) and Hoechst 33342 (blue) co-staining in macrophages. n = 6. M − N, Western blot and quantification of NLRP3, Cl-caspase-1, IL-1β, ASC and N-GSDMD protein levels in macrophages stimulated with LPS + ATP. n = 6. All data are mean ± SD. Statistical significance for panel E was determined by Student's t-test; all other quantitative data were analyzed by one-way ANOVA followed by Tukey's post-hoc test. ∗*P* < 0.05, ∗∗*P* < 0.01 vs. Con group; ^#^*P* < 0.05, ^##^*P* < 0.01 vs. LPS group.

To elucidate the mechanisms underlying excessive pulmonary inflammation, KEGG pathway analysis was first performed based on bulk RNA sequencing data. Among inflammation-related pathways, the NOD-like receptor signaling pathway was significantly enriched in LPS-induced ALI lungs (Fig. 3A). Subsequent heatmap analysis revealed marked upregulation of NLRP3 inflammasome-related proteins in LPS-treated lungs, including NLRP3, caspase-1, IL-1β, ASC, and the pyroptosis marker N-GSDMD (Fig. 3B), consistent with observations from an independent dataset (GSE263867) (Supplementary Fig. S3A). GSVA further indicated a significant correlation between TRPV4 expression and NLRP3 pathway activity in the lungs of LPS-treated mice (Fig. 3C).

To validate these transcriptomic findings at the protein level, Western blot analysis was performed on lung tissues. Compared with controls, LPS-challenged mice exhibited significantly increased protein expression of NLRP3, cleaved-caspase-1(Cl-caspase-1), IL-1β, N-GSDMD, and ASC (Fig. 3D and E). Immunofluorescence staining further demonstrated elevated expression of ASC and N-GSDMD in pulmonary macrophages of LPS-treated lungs (Fig. 3F and G and Supplementary Fig. S3B–C), indicating inflammasome activation and pyroptotic signaling in this cell population. Consistent with these findings, TEM revealed typical ultrastructural features of pyroptosis in macrophages, including cellular swelling, plasma membrane rupture, and cytoplasmic content release (Fig. 3H).

To further investigate whether excessive pulmonary inflammation is associated with NLRP3 inflammasome activation and pyroptosis in macrophages, an *in vitro* inflammatory model was established using macrophages stimulated with LPS plus ATP. ELISA showed that IL-1β and TNF-α concentrations in the culture supernatants were significantly increased in the LPS + ATP-treated group compared with controls (Fig. 3I). Morphological analysis by TEM revealed the characteristic "ballooning" phenotype and membrane rupture (Fig. 3J), while immunofluorescence images showed the translocation of N-GSDMD to the plasma membrane (Supplementary Fig. S3D–E). To distinguish pyroptosis from other forms of programmed cell death, we performed Annexin V/PI and PI/Hoechst flow cytometric analyses. The results showed a predominant Annexin V^+^/PI^+^ population (Supplementary Fig. S3F–I), confirming a lytic, pro-inflammatory death rather than non-inflammatory early apoptosis. Parallel protein analysis in both immortalized macrophages and primary BMDMs demonstrated a synchronized upregulation of TRPV4, the NLRP3 machinery, and N-GSDMD (Fig. 3M and N and Supplementary Fig. S3J–K). Finally, given that TRPV4 is a Ca^2+^-permeable channel, we used Fluo-4 AM imaging to monitor Ca^2+^ flux, revealing that the inflammatory milieu significantly enhanced intracellular Ca^2+^ accumulation (Supplementary Fig. S3L–M). Taken together, these multi-modal findings demonstrate that TRPV4 upregulation, concomitant with enhanced Ca^2+^ influx, is intrinsically linked to NLRP3 inflammasome activation and macrophage pyroptosis in the pathogenesis of ALI.

### 3.4 TRPV4 deficiency attenuates NLRP3 inflammasome activation and macrophage pyroptosis in excessive pulmonary inflammation during ALI

Given the prominent activation of the NLRP3 inflammasome and pyroptotic cell death observed in macrophages during LPS-induced ALI, we next examined whether TRPV4 deficiency could interrupt this inflammatory cascade. Lung tissues were harvested from LPS-challenged TRPV4^flox/flox^ and TRPV4^CKO^ mice for subsequent analyses.

**Fig. 4:**
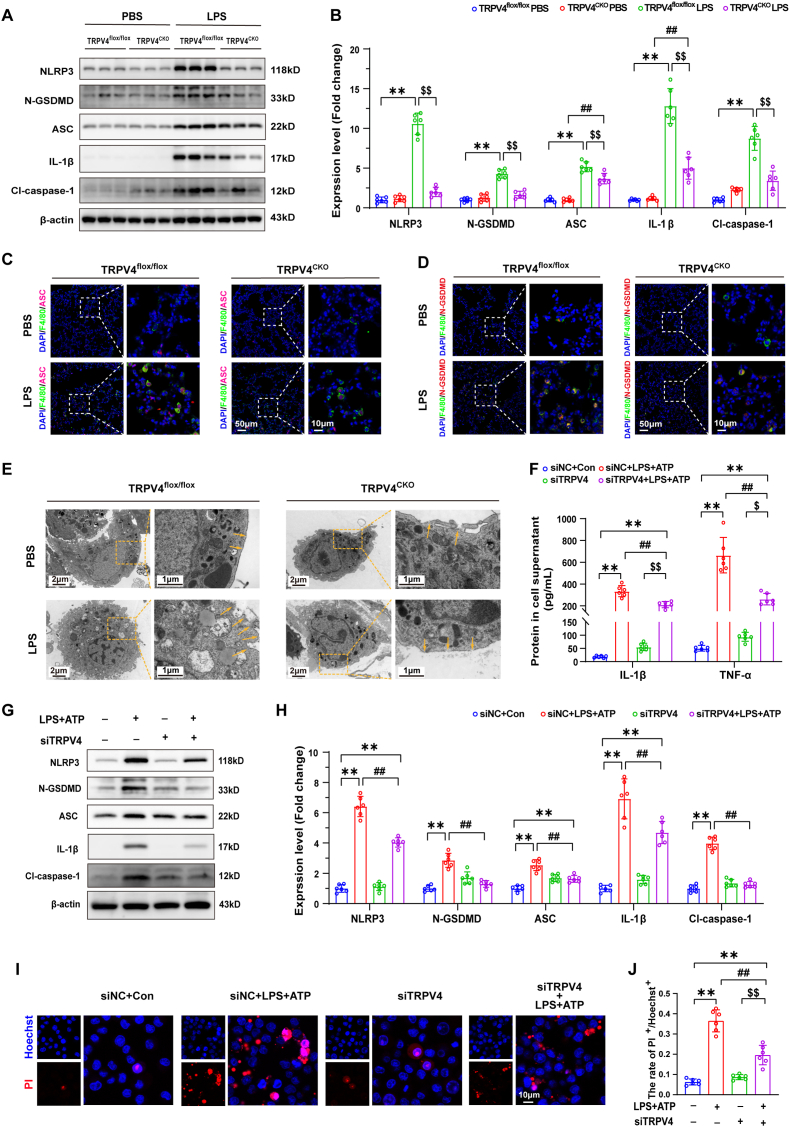
Myeloid cell-specific TRPV4 deficiency alleviates pulmonary inflammation by inhibiting NLRP3 inflammasome activation and macrophage pyroptosis A-B, Western blot analysis and quantification of NLRP3, Cl-caspase-1, IL-1β, ASC and N-GSDMD protein levels in lungs of TRPV4^flox/flox^ and TRPV4^CKO^ mice with PBS or LPS, n = 6. C-D, Representative immunofluorescence images exhibiting co-immunostaining of ASC (red) or N-GSDMD (red) with F4/80 (green) in lung sections of TRPV4^flox/flox^ and TRPV4^CKO^ mice treated with PBS or LPS. Scale bar: 50 μm and 10 μm. E, Representative TEM images of macrophages in lungs of TRPV4^flox/flox^ and TRPV4^CKO^ mice treated with PBS or LPS. Scale bar: 2 μm and 1 μm. F, Levels of IL-1β and TNF-α in the supernatants of cells transfected with siTRPV4 prior to LPS + ATP stimulation. n = 6. G-H, Western blot analysis and quantification of NLRP3, Cl-caspase-1, IL-1β, ASC and N-GSDMD protein levels in macrophages following transfected with siTRPV4 prior to LPS + ATP stimulation. n = 6. I-J, Representative immunofluorescence images and quantification of PI (red) and Hoechst 33342 (blue) co-staining in macrophages transfected with siTRPV4 and then stimulated with LPS + ATP. n = 6. All data are mean ± SD. Statistical significance was determined by One-way ANOVA followed by Tukey's post-hoc test. ∗*P* < 0.05, ∗∗*P* < 0.01 vs. TRPV4^flox/flox^ PBS or siNC + Con group; ^#^*P* < 0.05, ^##^*P* < 0.01 vs. TRPV4^CKO^ PBS or siNC + LPS + ATP group; ^$^*P* < 0.05, ^$$^*P* < 0.01 vs. TRPV4^flox/flox^ LPS or siTRPV4 group.

Genetic disruption of TRPV4 expression markedly attenuated inflammasome activation *in vivo*. Compared with LPS-treated TRPV4^flox/flox^ mice, TRPV4^CKO^ mice exhibited substantially reduced protein expression of NLRP3, Cl-caspase-1, mature IL-1β, ASC, and the pyroptotic effector N-GSDMD in lung tissues (Fig. 4A and B). Consistently, immunofluorescence staining of TRPV4^CKO^ lung tissues showed a reduction in ASC speck formation and reduced N-GSDMD expression in macrophages (Fig. 4C and D and Supplementary Fig. S4A–B). Ultrastructural examination further confirmed a marked attenuation of pyroptotic features in alveolar macrophages, as evidenced by reduced plasma membrane rupture and cytoplasmic content release in TRPV4-deficient mice (Fig. 4E).

The causal role of TRPV4 in regulating inflammasome-driven pyroptosis was further validated *in vitro*. Silencing of TRPV4 in macrophages using siRNA (Supplementary Fig. S4C–E), followed by LPS plus ATP stimulation, resulted in a pronounced suppression of inflammasome activation. Specifically, TRPV4 knockdown significantly reduced the release of IL-1β and TNF-α into the culture supernatants (Fig. 4F) and markedly decreased the expression of NLRP3, Cl-caspase-1, IL-1β, N-GSDMD, and ASC (Fig. 4G and H). This molecular inhibition was further corroborated by immunofluorescence analysis, showing a significant reduction in N-GSDMD MFI (Supplementary Fig. S4F–G). Moreover, both immunofluorescence imaging and flow cytometric analysis of PI/Hoechst staining demonstrated that TRPV4 silencing significantly rescued macrophages from lytic death, as indicated by the decreased proportion of PI-positive cells (Fig. 4I and J and Supplementary Fig. S4H–I). Collectively, this *in vivo* and *in vitro* genetic evidence firmly establish that TRPV4 acts as a requisite upstream regulator of NLRP3 inflammasome activation and subsequent macrophage pyroptosis, thereby fueling the excessive inflammatory milieu during ALI.

### 3.5 TRPV4 activation disrupts mitochondrial homeostasis and energetic status in macrophages

**Fig. 5:**
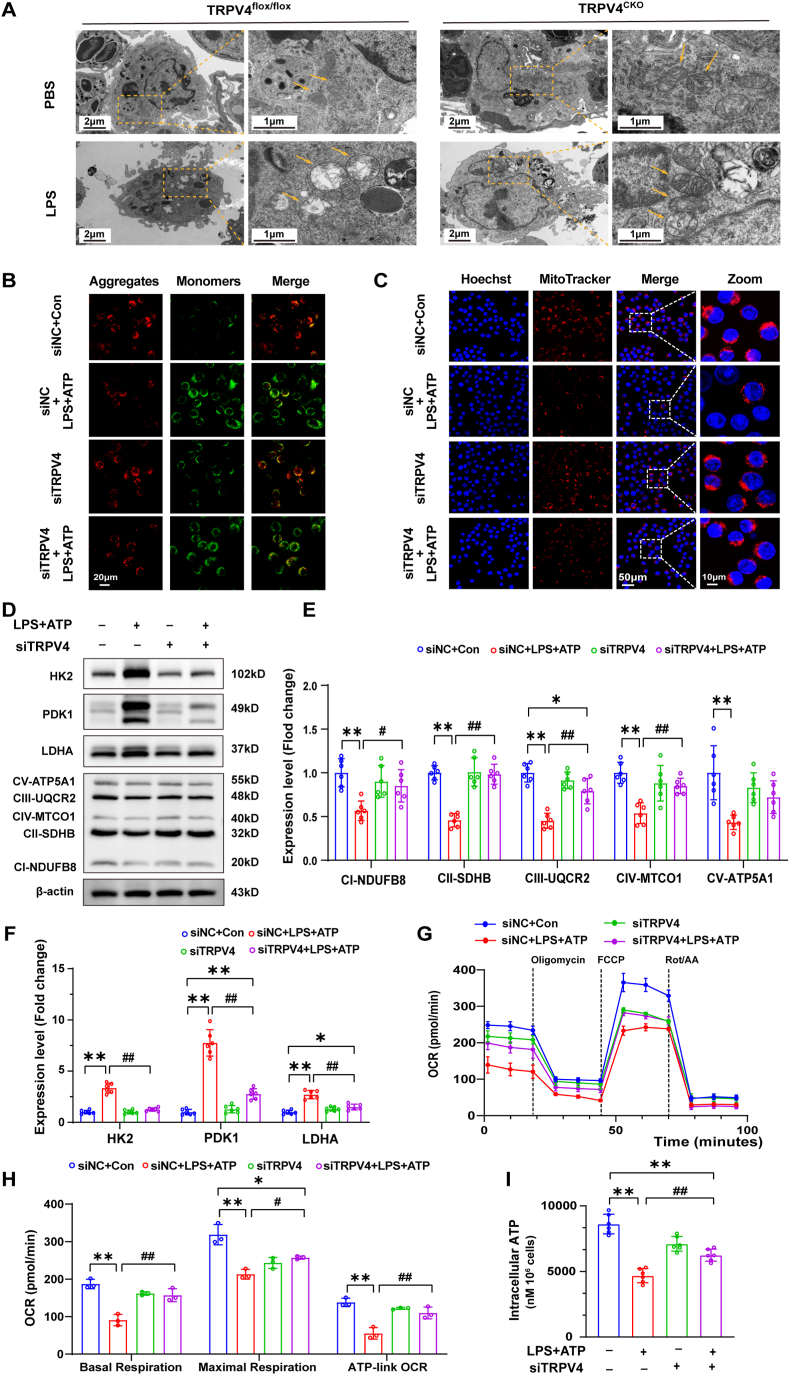
TRPV4 knockdown restores mitochondrial function in inflamed macrophages A, Representative TEM images of macrophages in lungs of TRPV4^flox/flox^ and TRPV4^CKO^ mice treated with PBS or LPS. Scale bar: 2 μm and 1 μm. B, Representative images of JC-1 staining in macrophages transfected with siTRPV4 prior to LPS + ATP stimulation. Scale bar: 20 μm. C, Representative images of MitoTracker staining in macrophages transfected with siTRPV4 prior to LPS + ATP stimulation. Scale bar: 50 μm and 10 μm. D-F, Western blot analysis and quantification of OXPHOS-complex related proteins (NADH: ubiquinone oxidoreductase subunit B8 (NDUFB8), succinate dehydrogenase complex iron-sulfur subunit B (SDHB), ubiquinol-cytochrome c reductase core protein 2 (UQCR2), mitochondrially encoded cytochrome *c* oxidase I (MTCO1), ATP synthase F1 subunit alpha (ATP5A1)) and glycolytic enzymes (hexokinase 2 (HK2), pyruvate dehydrogenase kinase 1(PDK1) and lactate dehydrogenase A (LDHA)) in macrophages transfected with siTRPV4 prior to LPS + ATP stimulation. n = 6. G-H, Seahorse profiles and quantification of oxygen consumption rate (OCR) in macrophages following transfection with siTRPV4 prior to LPS + ATP stimulation. n = 3. I, ATP activity in macrophages following transfection with siTRPV4 prior to LPS + ATP stimulation. n = 6. All data are mean ± SD. Statistical significance was determined by One-way ANOVA followed by Tukey's post-hoc test. ∗*P* < 0.05, ∗∗*P* < 0.01 vs. siNC + Con group; ^#^*P* < 0.05, ^##^*P* < 0.01 vs. siNC + LPS + ATP group.

Accumulating evidence indicates that mitochondrial dysfunction represents a critical upstream signal for NLRP3 inflammasome activation [13,15,25,33]. To understand whether TRPV4 contributes to this pathway, we assessed its role in regulating mitochondrial function. We first examined mitochondrial ultrastructural alterations in lung tissues using TEM. The results demonstrated that genetic deletion of TRPV4 markedly alleviated mitochondrial ultrastructural damage in pulmonary macrophages, as evidenced by reduced mitochondrial swelling and preservation of cristae architecture (Fig. 5A and Supplementary Fig. S5A–D). Subsequently, *in vitro* experiments were conducted to further investigate whether TRPV4 deficiency improves mitochondrial homeostasis in macrophages upon inflammatory stimulation. In macrophages stimulated with LPS plus ATP, TRPV4 silencing significantly restored mitochondrial function. JC-1 staining revealed a pronounced improvement in mitochondrial membrane potential, as indicated by an increased red-to-green fluorescence ratio following TRPV4 knockdown (Fig. 5B and Supplementary Fig. S5E). In parallel, MitoTracker staining demonstrated a significant reduction in the proportion of morphologically abnormal mitochondria, indicating preservation of mitochondrial structural integrity upon TRPV4 silencing (Fig. 5C and Supplementary Fig. S5F).

At the protein and functional levels, TRPV4 knockdown markedly improved mitochondrial energy metabolism under inflammatory conditions. Immunoblotting analyses showed that the protein expression levels of multiple oxidative phosphorylation (OXPHOS) complex components, including MTCO1, UQCRC2, SDHB, and NDUFB8, were significantly increased in inflamed macrophages following TRPV4 silencing (Fig. 5D and E). Concurrently, the expression of key glycolytic enzymes, such as HK2, PDK1, and LDHA, was significantly reduced (Fig. 5F). Consistent with these molecular alterations, Seahorse extracellular flux analysis revealed that TRPV4 knockdown significantly reversed the inflammation-induced reduction in mitochondrial basal respiration, maximal respiration, and ATP-linked oxygen consumption rates in macrophages (Fig. 5G and H). Notably, intracellular ATP levels were significantly elevated following TRPV4 silencing (Fig. 5I), indicating a substantial improvement in overall mitochondrial energetic status. These findings indicate that TRPV4 deficiency alleviates mitochondrial dysfunction in inflamed macrophages, raising the possibility that specific mitochondrial-derived signals may link TRPV4 activation to downstream inflammasome activation.

### 3.6 TRPV4 exacerbates NLRP3 inflammasome activation and macrophage pyroptosis through Ca^2+^-dependent mitochondrial dysfunction and mtROS production

To determine whether Ca^2+^ influx is required for TRPV4-mediated mitochondrial impairment, macrophages were pretreated with the intracellular Ca^2+^ chelator BAPTA prior to stimulation. As expected, TRPV4 activation by GSK triggered a robust elevation in cytosolic Ca^2+^ (Supplementary Fig. S6A and D), an effect that was effectively abolished by BAPTA, thereby validating the efficacy of Ca^2+^ chelation in our model.

**Fig. 6:**
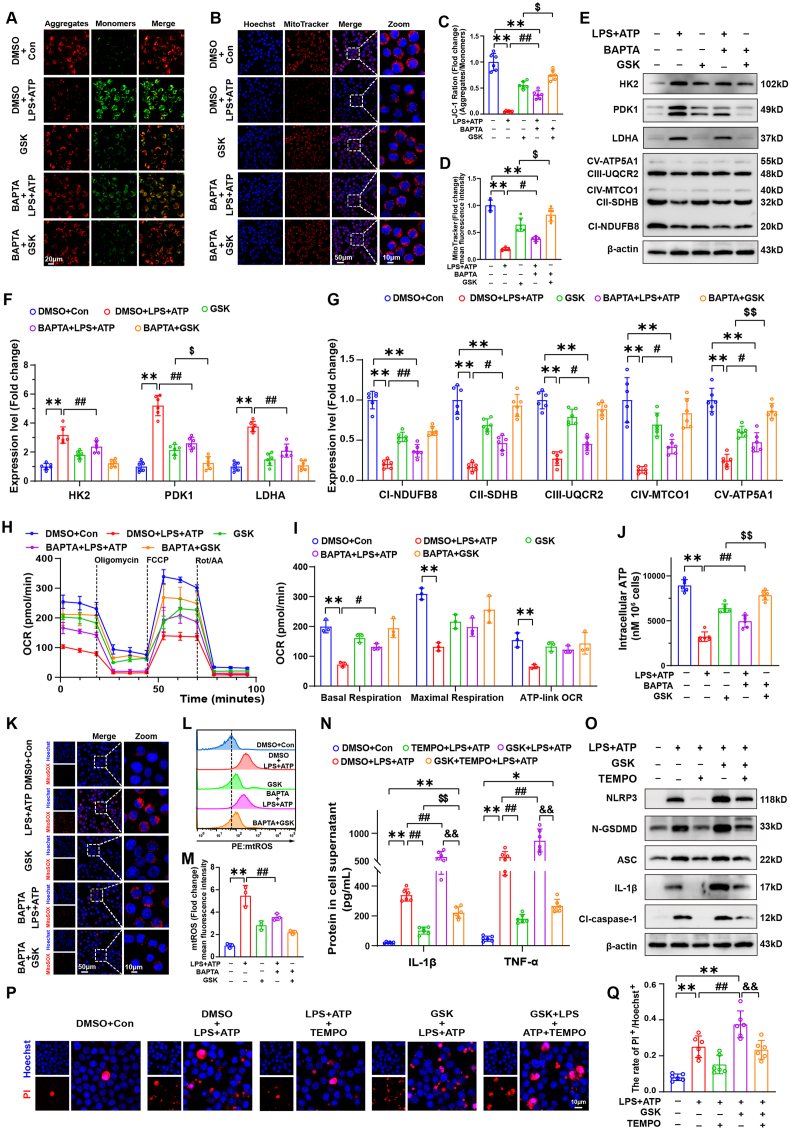
TRPV4-mediated Ca^2+^ influx drives mitochondrial dysfunction and mtROS-dependent NLRP3 inflammasome activation in macrophages A–D, Representative fluorescence images and quantification of JC-1 staining (A, C) and MitoTracker staining (B, D) in macrophages pretreated with or without BAPTA or GSK, followed by LPS + ATP stimulation. JC-1 red-to-green ratio reflects mitochondrial membrane potential. n = 6 (C–D). Scale bars: 20 μm (A); 50 μm and 10 μm (insets, B). E-G, Western blot analysis and quantification of OXPHOS-complex related proteins (ATP5A1, MTCO1, UQCRC2, SDHB, NDUFB8) and glycolytic enzymes (HK2, PDK1, LDHA) in macrophages pretreated with or without BAPTA or GSK, followed by LPS + ATP stimulation. n = 6. H–I, Seahorse profiles and quantification of oxygen consumption rate (OCR) in macrophages pretreated with or without BAPTA or GSK, followed by LPS + ATP stimulation. n = 3. J, ATP activity in macrophages pretreated with or without BAPTA or GSK, followed by LPS + ATP stimulation. n = 6. K-M, mtROS production assessed by MitoSOX staining (K) and flow cytometry (L, M). Scale bars: 50 μm and 10 μm (insets, K). Flow cytometry (L) and quantification (M) of mtROS in macrophages pretreated with or without BAPTA or GSK, followed by LPS + ATP stimulation. n = 3. N–O, NLRP3 inflammasome activation and pyroptosis in macrophages, including IL-1β and TNF-α levels in supernatants (N), Western blot analysis of NLRP3, ASC, Cl-caspase-1, IL-1β, and N-GSDMD (O). n = 6. P-Q, Representative immunofluorescence images and quantification of PI (red) and Hoechst 33342 (blue) co-staining in macrophages pretreated with or without BAPTA or GSK, followed by LPS + ATP stimulation. n = 6. All data are mean ± SD. Statistical significance was determined by One-way ANOVA followed by Tukey's post-hoc test. ∗*P* < 0.05, ∗∗*P* < 0.01 vs. DMSO + Con group; ^#^*P* < 0.05, ^##^*P* < 0.01 vs. DMSO + LPS + ATP group; ^$^*P* < 0.05, ^$$^*P* < 0.01 vs. GSK or TEMPO + LPS + ATP group; ^&^*P* < 0.05, ^&&^*P* < 0.01 vs. GSK + LPS + ATP group.

Notably, the integrity of the mitochondrial network under TRPV4 activation was strictly Ca^2+^-dependent. JC-1 staining demonstrated that the dissipation of mitochondrial membrane potential induced by GSK was significantly rescued by BAPTA pretreatment (Fig. 6A, C and Supplementary Fig. S6B and E). Furthermore, MitoTracker imaging revealed that the marked mitochondrial fragmentation and morphological abnormalities triggered by TRPV4 were substantially attenuated upon Ca^2+^ chelation (Fig. 6 B, D and Supplementary Fig. S6C and F).

At the bioenergetic level, TRPV4 activation severely compromised mitochondrial OXPHOS, evidenced by the downregulated expression of key electron transport chain subunits, including ATP5A1, MTCO1, UQCRC2, SDHB, and NDUFB8 (Supplementary Fig. S6G and H). Conversely, BAPTA partially restored mitochondrial proteostasis, specifically rescuing the levels of ATP5A1, MTCO1, UQCRC2, SDHB, and NDUFB8 (Fig. 6E–G). This mitochondrial suppression was accompanied by a shift toward glycolytic reprogramming, characterized by the upregulation of HK2, PDK1, and LDHA, a metabolic rewiring that was significantly blunted by Ca^2+^ chelation (Supplementary Fig. S6G–H and Fig. 6E and F). Seahorse extracellular flux analysis further demonstrated that TRPV4 activation suppressed mitochondrial respiration, including basal respiration, maximal respiration, and ATP-linked respiration (Supplementary Fig. S6I–J), while BAPTA pretreatment significantly rescued basal respiration (Fig. 6H and I) and restored intracellular ATP pools (Fig. 6J). Together, these data establish that TRPV4-driven mitochondrial dysfunction is an obligatory consequence of Ca^2+^ influx.

It is well established that mtROS serve as critical mediators of NLRP3 inflammasome activation, ranking among the most pivotal mitochondria-derived signals [[36], [37], [38]]. Accordingly, we next examined whether mitochondrial dysfunction induced by TRPV4 activation was accompanied by excessive mtROS production. Both MitoSOX staining and flow cytometry revealed a massive accumulation of mtROS in GSK-treated macrophages under inflammatory priming (Supplementary Fig. S6L–O). Crucially, this oxidative burst was significantly quenched by BAPTA (Fig. 6K–M; Supplementary Fig. S6P), identifying Ca^2+^ as the upstream driver of mtROS generation in this context.

To define the functional requirement of mtROS in linking TRPV4 to the inflammasome, we employed the mtROS-targeted scavenger TEMPO. Scavenging mtROS markedly suppressed inflammatory secretome, as evidenced by reduced titers of IL-1β and TNF-α (Fig. 6N). At the molecular level, TEMPO treatment substantially inhibited the assembly and activation of the NLRP3 inflammasome, reflected by the diminished Cl-caspase-1, N-GSDMD, and mature IL-1β expression (Fig. 6O and Supplementary Fig. S7A).

Consistent with the inhibition of GSDMD-mediated pore formation, N-GSDMD immunosignals were significantly attenuated upon mtROS scavenging, with a marked loss of its distinctive membrane localization (Supplementary Fig. S7B–C). Functionally, the executive phase of cell death–pyroptosis–was largely prevented by TEMPO, as indicated by a significant reduction in the proportion of PI^+^/Hoechst^+^ macrophages (Fig. 6P and Q and Supplementary Fig. S7D–E).

In summary, our findings support a mechanistic model in which TRPV4-mediated Ca^2+^ influx precipitates mitochondrial bioenergetic collapse and mtROS hyperproduction, which in turn serves as the critical metabolic signal driving NLRP3 inflammasome activation and subsequent macrophage pyroptosis.

### 3.7 Pharmacological inhibition of TRPV4 by HC-067047 abolishes NLRP3 inflammasome activation and macrophage pyroptosis *in vitro*

**Fig. 7:**
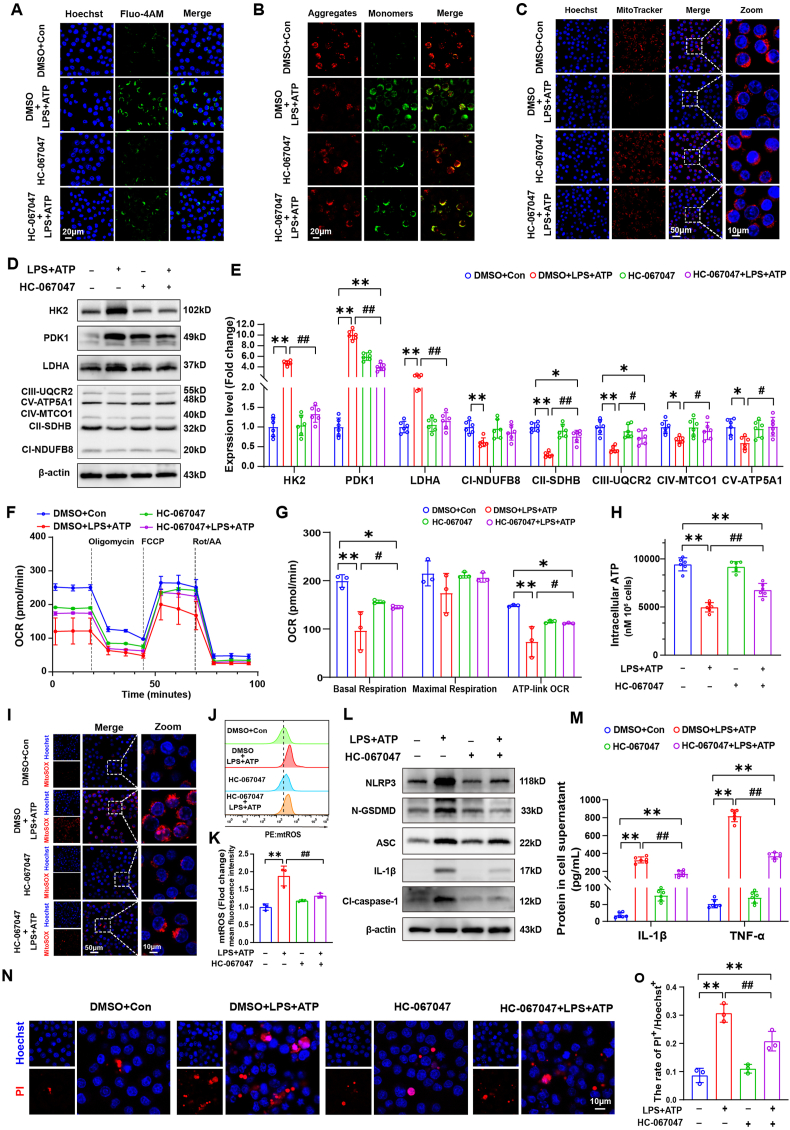
Pharmacological inhibition of TRPV4 by HC-067047 suppresses NLRP3 inflammasome activation and macrophage pyroptosis *in vitro* A, Representative images of Fluo-4AM staining in macrophages pretreated with or without HC-067047 followed by stimulation with LPS + ATP. Scale bar: 20 μm. B, Representative images of JC-1 staining in macrophages pretreated with or without HC-067047 followed by stimulation with LPS + ATP. Scale bar: 20 μm. C, Representative images of MitoTracker staining in macrophages pretreated with or without HC-067047 followed by stimulation with LPS + ATP. Scale bar: 50 μm and 10 μm. D-E, Western blot and quantification of ATP5A1, MTCO1, UQCRC2, SDHB, NDUFB8, HK2, PDK1 and LDHA in macrophages pretreated with or without HC-067047 followed by stimulation with LPS + ATP. n = 6. F-G, Seahorse profiles and quantification of OCR in macrophages pretreated with or without HC-067047 followed by stimulation with LPS + ATP. n = 3. H, ATP activity in macrophages pretreated with or without HC-067047 followed by stimulation with LPS + ATP. n = 6. I, Representative images of MitoSOX staining in macrophages pretreated with or without HC-067047 followed by stimulation with LPS + ATP. Scale bar: 50 μm and 10 μm. J-K, Flow cytometry and quantification of mtROS in macrophages pretreated with or without HC-067047 followed by stimulation with LPS + ATP. n = 3. L, Representative images of Western blot of NLRP3, Cl-caspase-1, IL-1β, ASC and N-GSDMD protein levels in macrophages pretreated with or without HC-067047 followed by stimulation with LPS + ATP. n = 6. M, IL-1β and TNF-α levels in the supernatants pretreated with or without HC-067047 followed by stimulation with LPS + ATP. n = 6. N–O, Representative immunofluorescence images and quantification of PI (red) and Hoechst 33342 (blue) co-staining in macrophages pretreated with or without HC-067047 followed by stimulation with LPS + ATP. n = 6. All data are mean ± SD. Statistical significance was determined by One-way ANOVA followed by Tukey's post-hoc test. ∗*P* < 0.05, ∗∗*P* < 0.01 vs. DMSO + Con group; ^#^*P* < 0.05, ^##^*P* < 0.01 vs. DMSO + LPS + ATP group.

We next investigated whether pharmacological blockade of TRPV4 could recapitulate the protective effects on mitochondrial homeostasis in inflamed macrophages. HC-067047, a benzothiophene-derived TRPV4 antagonist that binds to the S1–S4 region of the channel, was employed to inhibit TRPV4 activity [39]. Fluo-4 AM staining demonstrated that HC-067047 effectively suppressed TRPV4-dependent Ca^2+^ influx, as evidenced by a marked reduction in fluorescence intensity (Fig. 7A and Supplementary Fig. S8A). Under inflammatory conditions, pharmacological inhibition of TRPV4 was sufficient to restore mitochondrial homeostasis. Treatment with HC-067047 significantly improved mitochondrial membrane potential and reduced the accumulation of morphologically abnormal mitochondria in LPS + ATP-stimulated macrophages (Fig. 7B and C and Supplementary Fig. S8B–C).

In addition, TRPV4 blockade markedly corrected inflammation-induced dysregulation of mitochondrial protein expression. Specifically, the protein levels of OXPHOS complex components, including ATP5A1, MTCO1, UQCRC2, SDHB, and NDUFB8, were increased, whereas the expression of glycolysis-associated enzymes, including HK2, PDK1, and LDHA, was reduced (Fig. 7D and E). These molecular alterations were accompanied by improved mitochondrial respiratory capacity. Seahorse metabolic flux analysis indicated that TRPV4 inhibition reversed the inflammation-induced reductions in mitochondrial basal respiration and ATP-linked oxygen consumption rates in macrophages (Fig. 7F and G). The concomitant increase in intracellular ATP levels further substantiated these findings (Fig. 7H).

Consistent with restored mitochondrial function, TRPV4 inhibition significantly attenuated mtROS accumulation (Fig. 7I–K and Supplementary Fig. S8D), thereby uncoupling mitochondrial stress from downstream inflammasome signaling. Accordingly, HC-067047 treatment markedly suppressed NLRP3 inflammasome activation (Fig. 7L and Supplementary Fig. S8E), reduced the release of pro-inflammatory cytokines (Fig. 7M). Consistent with the inhibition of the pyroptotic cascade, immunofluorescence analysis revealed a significant reduction in N-GSDMD fluorescence intensity upon HC-067047 treatment, indicating a suppression of N-GSDMD-mediated membrane pore formation (Supplementary Fig. S8F–G). This molecular protection translated into enhanced cell viability; both PI/Hoechst co-staining and flow cytometry demonstrated a marked decrease in the proportion of pyroptotic (PI^+^) cells (Fig. 7N and O and Supplementary Fig. S8H–I). Collectively, these findings indicate that pharmacological inhibition of TRPV4 recapitulates the protective effects of genetic TRPV4 silencing, thereby suppressing mitochondrial dysfunction–driven NLRP3 inflammasome activation and macrophage pyroptosis *in vitro*.

### 3.8 Pharmacological inhibition of TRPV4 with HC-067047 alleviates pulmonary inflammation and lung injury *in vivo*

**Fig. 8:**
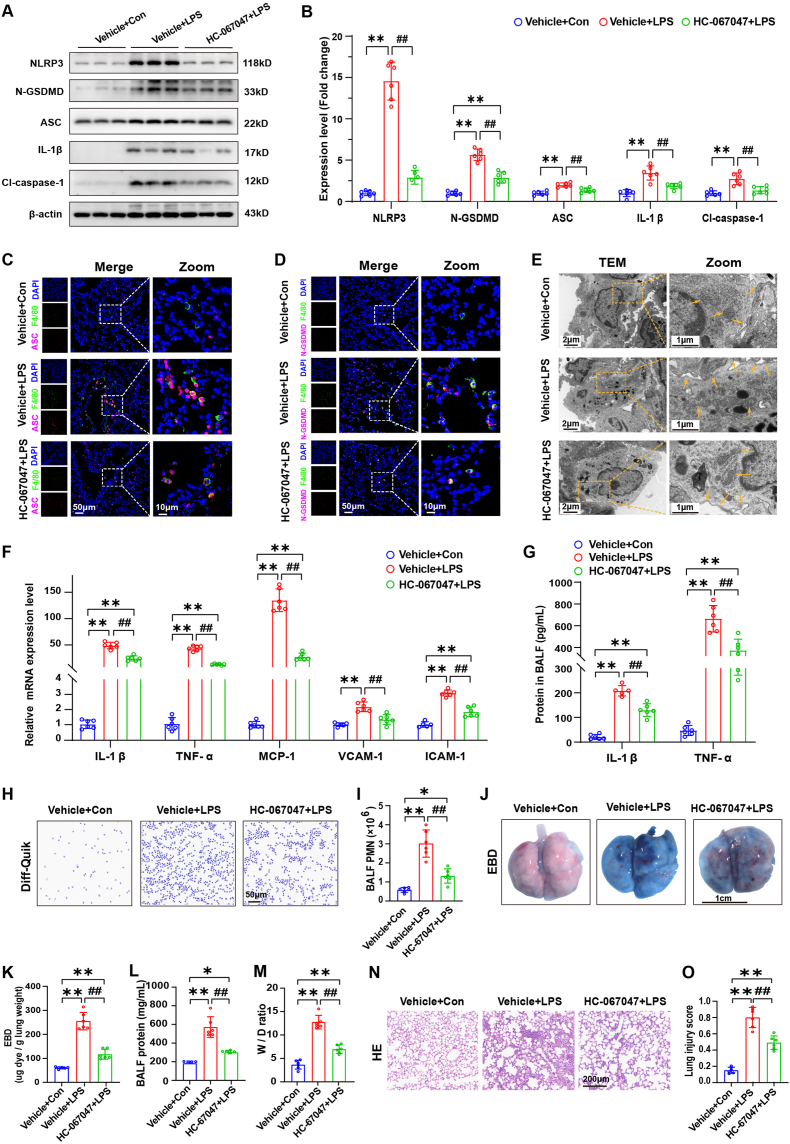
Pharmacological blockade of TRPV4 with HC-06704 suppresses inflammatory amplification and lung injury *in vivo* A-B, Western blot analysis and quantification of NLRP3, Cl-caspase-1, IL-1β, ASC and N-GSDMD protein levels in lungs of mice pretreated with or without HC-067047 followed by LPS, n = 6. C, Representative immunofluorescent images exhibiting co-immunostaining of ASC (shown in red) with F4/80 (shown in green) in lung sections of mice with or without HC-067047 followed by LPS or PBS. Scale bar: 50 μm and 10 μm. D, Representative immunofluorescent images exhibiting co-immunostaining of N-GSDMD (shown in red) with F4/80 (shown in green) in the lung sections of mice with or without HC-067047 followed by LPS or PBS. Scale bar: 50 μm and 10 μm. E, Representative TEM images of macrophages in the lungs of mice with or without HC-067047 followed by LPS or PBS. Scale bar: 2 μm and 1 μm. F, Expression of IL-1β, TNF-α, MCP-1, VCAM-1 and ICAM-1 mRNA in lungs of mice with or without HC-067047 followed by LPS or PBS. n = 6. G, Levels of IL-1β and TNF-α in the BALF of mice with or without HC-067047 followed by LPS or PBS. n = 6. H, Representative macroscopic images of BALF cytospin preparations from mice with or without HC-067047 followed by LPS or PBS, stained with Diff-Quik. Scale bar: 50 μm. I, PMNs count in BALF from mice treated with or without HC-067047 followed by LPS or PBS, determined by Diff-Quik staining. n = 6. J-K, Representative images of lung stained with EBD in lungs of mice with or without HC-067047 followed by LPS or PBS. Scale bar: 1 cm. L, Protein concentrations in the BALF of mice with or without HC-067047 followed by LPS or PBS. n = 6. M, Lung W/D ratio of mice with or without HC-067047 followed by LPS or PBS. n = 6. N, Representative macroscopic images of the lung sections stained with HE of mice with or without HC-067047 followed by LPS or PBS. scale bar: 200 μm. O, Quantification of pathological lung injury scores based on HE staining. n = 6. All data are mean ± SD. Statistical significance was determined by one-way ANOVA followed by Tukey's post-hoc test. *∗P* < 0.05, ∗∗*P* < 0.01 vs. Vehicle + Con group; ^#^*P* < 0.05, ^##^*P* < 0.01 vs. Vehicle + LPS group.

Having demonstrated that TRPV4 blockade exerts robust anti-inflammatory and anti-pyroptotic effects at the cellular level, we next evaluated the efficacy of the pharmacological inhibitor HC-067047 in protecting against ALI *in vivo*. Compared with LPS-treated mice, administration of HC-067047 markedly suppressed activation of the NLRP3 inflammasome in lung tissues, as evidenced by reduced expression of NLRP3, Cl-caspase-1, ASC, IL-1β, and N-GSDMD (Fig. 8A and B). Consistently, immunofluorescence analyses demonstrated decreased ASC and N-GSDMD expression in macrophages following HC-067047 treatment (Fig. 8C and D and Supplementary Fig. S9A–B). Ultrastructural examination by TEM further revealed that TRPV4 blockade alleviated macrophage pyroptotic features, including reduced plasma membrane rupture and cytoplasmic content release, accompanied by a marked improvement in mitochondrial ultrastructure, as reflected by attenuated mitochondrial swelling and preservation of cristae architecture (Fig. 8E). At the tissue level, HC-067047 treatment significantly dampened pulmonary inflammatory responses, as indicated by reduced mRNA expression of IL-1β, TNF-α, ICAM-1, MCP-1, and VCAM-1 in lung homogenates (Fig. 8F), together with decreased concentrations of IL-1β and TNF-α in BALF (Fig. 8G). In parallel with the resolution of inflammation, pulmonary microvascular barrier integrity was substantially restored. TRPV4 inhibition led to a significant reduction in neutrophil infiltration into the alveolar space (Fig. 8H and I). Moreover, the leakage of plasma constituents was effectively blunted, as evidenced by decreased Evans blue extravasation, lower BALF protein concentrations, and a reduced lung W/D weight ratio (Fig. 8J–M). Finally, histopathological assessment via HE staining confirmed that pharmacological TRPV4 blockade attenuated alveolar septal thickening, preserved alveolar airspace, and diminished inflammatory cell sequestration, culminating in a significant reduction in the overall lung injury score (Fig. 8N and O). Collectively, these *in vivo* findings demonstrate that pharmacological TRPV4 blockade by HC-067047 alleviates inflammasome-driven pulmonary inflammation and injury. The close concordance between *in vivo* pharmacological inhibition, genetic TRPV4 silencing, and *in vitro* findings supports a unified model in which TRPV4-mediated mitochondrial dysfunction and downstream NLRP3 inflammasome activation in macrophages drive excessive pulmonary inflammation.

## 4 Discussion

This study identifies TRPV4 as a pivotal upstream regulator that links Ca^2+^ influx to mitochondrial dysfunction, thereby driving NLRP3 inflammasome activation and macrophage pyroptosis during ALI. By integrating *in vivo* genetic models, pharmacological inhibition, and *in vitro* mechanistic analyses, our data support a model in which TRPV4-dependent mitochondrial stress functions as a key intermediary connecting extracellular inflammatory cues to intracellular inflammasome activation, positioning TRPV4 as a mechanistic driver and a potential therapeutic target in ALI/ARDS.

TRPV4 is a non-redundant Ca^2+^ entry pathway in macrophages, and previous studies have implicated it in pulmonary inflammation through vascular and epithelial effects. However, its cell-intrinsic function in innate immune cells remains incompletely defined [[40], [41], [42]]. Our findings substantially extend the current understanding of TRPV4 biology by identifying pulmonary macrophages as a critical cellular context in which TRPV4 exerts a mitochondria-centered pro-inflammatory function. Rather than acting solely as an upstream Ca^2+^ conduit, TRPV4 emerges as a regulator of mitochondrial integrity and redox homeostasis in macrophages. TRPV4 activation induces a decrease in mitochondrial membrane potential, ultrastructural damage, impaired OXPHOS, and excessive mtROS accumulation, collectively indicating severe mitochondrial dysfunction. These results position mitochondrial stress as an early and central downstream consequence of TRPV4 activation in macrophages during ALI.

A central implication of this study is the TRPV4–Ca^2+^–mitochondrial axis that governs inflammasome activation [[43], [44], [45]]. Consistent with established evidence that mitochondrial dysfunction drives NLRP3 activation, we show that TRPV4-induced Ca^2+^ influx leads to mitochondrial impairment and excessive mtROS production, which promotes inflammasome activation and pyroptotic cell death. Importantly, pharmacological scavenging of mtROS markedly attenuated inflammasome activation, functionally positioning mtROS as a necessary signaling intermediary rather than a downstream byproduct.

Beyond its role in inflammatory signaling, TRPV4 also regulates mitochondrial bioenergetic homeostasis under basal conditions. TRPV4 silencing reduced oxygen consumption and ATP-linked respiration without altering mitochondrial respiratory chain protein expression, indicating functional modulation of mitochondrial activity rather than changes in electron transport chain abundance. This phenotype may be mediated by Ca^2+^-dependent regulation of tricarboxylic acid cycle enzyme activity [46], as supported by the similar effects observed following intracellular Ca^2+^ chelation with BAPTA-AM. Notably, the baseline fluctuations in absolute maximal respiratory capacity observed between independent experiments likely reflect the intrinsic sensitivity of macrophages to culture-dependent variables (such as cell passage and attachment efficiency), a common feature in real-time metabolic assays. Crucially, these variations did not alter the directionality of TRPV4-dependent effects. Under LPS/ATP stimulation, TRPV4 deficiency consistently attenuated the suppression of basal respiration across all replicates, indicating that TRPV4 specifically modulates mitochondrial vulnerability under acute inflammatory stress rather than dictating resting metabolic capacity. While TRPV4 inhibition modestly lowers this basal metabolic set-point, it simultaneously bolsters mitochondrial adaptability by reducing susceptibility to calcium overload and mtROS-driven failure during inflammatory stress. Together, these findings demonstrate that despite baseline fluctuations, TRPV4 robustly shapes the magnitude of inflammation-induced metabolic impairment by regulating mitochondrial susceptibility to inflammatory stress. In addition, TRPV4 inhibition was associated with the unexpected upregulation of PDK1 expression, which may reflect a secondary, compensatory adaptive response to altered cellular energetic status following mitochondrial functional modulation, rather than a direct transcriptional effect. Ultimately, these findings highlight that while TRPV4 influences baseline metabolic flux via Ca^2+^ dynamics, its primary physiological significance lies in setting the threshold for mitochondrial adaptation, thereby determining the degree to which mitochondrial function becomes compromised under inflammatory stress.

In addition to the mtROS-dependent mechanism identified in this study, mitochondrial DNA (mtDNA) release is recognized as a potent danger signal that can directly activate the NLRP3 inflammasome [47,48]. Although we did not directly quantify mtDNA, the profound decrease in mitochondrial membrane potential and structural damage observed following TRPV4 activation suggest that mtDNA leakage is a likely concurrent or downstream event. Such leakage may be facilitated by the transient or persistent opening of the mitochondrial permeability transition pore, a process known to be sensitive to calcium overload. Given that mtROS is often required for mtDNA oxidation and its subsequent immunogenicity, these two mitochondrial DAMPs may act synergistically to amplify the pyroptotic response, a possibility that warrants further dedicated investigation.

Broadly, our unravelling of this mitochondria-centered signaling pathway aligns with an emerging paradigm in which TRP channels function as integrators of environmental and intracellular stress signals in innate immunity. While channels such as TRPM2 [49] and TRPM7 [50] have been implicated in oxidative stress sensing and metabolic regulation [51], respectively, our findings define a distinct role for TRPV4 as a Ca^2+^-permeable conduit that functionally couples extracellular mechanical and inflammatory cues to mitochondrial dysfunction and mtROS-dependent inflammasome activation. In this context, TRPV4 establishes a direct mechanistic link between environmental sensing and immunometabolic reprogramming, thereby expanding the functional landscape of TRP channels in innate immunity.

Beyond its mechanistic implications, this study provides important translational insight by demonstrating that pharmacological inhibition of TRPV4 confers robust protection against inflammasome-driven lung injury. Using the selective TRPV4 antagonist HC-067047, we show that TRPV4 blockade markedly alleviates pulmonary vascular leakage, suppresses inflammatory cytokine production, reduces neutrophil infiltration, and improves histopathological lung injury in LPS-induced ALI. Notably, the overall protective profile of TRPV4 inhibition closely mirrors that observed in myeloid-specific TRPV4-deficient mice, underscoring the therapeutic relevance of targeting TRPV4 signaling in innate immune cells. Given that TRPV4 antagonists have already been explored in preclinical and early clinical settings for pulmonary and cardiovascular diseases [52,53], these findings support the feasibility of TRPV4 inhibition as a therapeutic strategy to limit excessive inflammation and tissue damage in ALI/ARDS.

Several limitations of this study should be acknowledged without detracting from the robustness of our core findings. First, while our Ca^2+^ chelation experiments identify ion influx as a requisite intermediate, the precise organelle-specific coupling, such as the role of the mitochondrial calcium uniporter or the modulation of mitochondrial dynamics [54], remain to be fully dissected. Nonetheless, our data support a functionally sufficient pathway whereby TRPV4-driven Ca^2+^ overload precipitates mitochondrial bioenergetic failure and mtROS hyperproduction. Second, the *Lyz2-Cre* transgenic model targets a broad myeloid spectrum, including neutrophils. Thus, our *in vivo* observations are appropriately interpreted as myeloid-driven. While our *in vitro* data demonstrate a cell-autonomous role for TRPV4 in macrophages, future studies utilizing more restricted Cre lines (e.g., *Adgre1-Cre*) [55] are warranted to further dissect the relative contributions of specific myeloid subsets in ALI. Third, this study focused on the early inflammatory phase (6 h) of LPS-induced ALI to capture the initiation of the TRPV4-mediated pyroptotic cascade. Therefore, the persistence of this signaling pathway during later stages of inflammation, resolution, or tissue remodeling remains unknown. In addition, the applicability of the TRPV4–Ca^2+^–mtROS–NLRP3 pathway to other clinically relevant inflammatory contexts, such as cytokine-driven inflammation, hyperoxic injury, or mechanical stretch-induced lung injury, has yet to be established. Nevertheless, because mtROS is widely recognized as a common upstream danger signal involved in NLRP3 inflammasome activation across diverse forms of tissue injury, this signaling axis may have broader relevance. Future studies employing extended observation periods and alternative injury models will be valuable to further evaluate the temporal persistence and generalizability of the mechanism identified here. Finally, although pharmacological inhibition of TRPV4 demonstrated clear protective effects in the acute setting, the long-term safety and efficacy of sustained TRPV4 blockade require careful evaluation before clinical translation.

In summary, this work supports a model in which TRPV4 acts as a critical molecular integrator that couples extracellular inflammatory cues to mitochondrial danger signaling in macrophages. By linking Ca^2+^ influx to mitochondrial dysfunction, mtROS accumulation, and inflammasome licensing, TRPV4 amplifies macrophage pyroptosis and sustains inflammatory escalation during ALI (Graphical abstract). These findings highlight ion channel-mitochondrial crosstalk as an important regulatory layer of innate immune responses in the lung and underscore mitochondrial stress-inflammasome coupling as a rational therapeutic axis for limiting excessive inflammation in ALI/ARDS.

## Ethics approval and consent to participate

All animal experiments were approved by the Institutional Animal Care and Use Committee of Beijing Anzhen Hospital, Capital Medical University, and were conducted in accordance with relevant institutional and national guidelines for the care and use of laboratory animals (approval no. AZ2025LA003).

## Consent for publication

Not applicable.

## Funding

This work was supported by the Chinese Institutes for Medical Research, Beijing (Grant CX24PY21), the R&D Program of Beijing Municipality Education Commission (Grant KZ202210025031) and the 10.13039/501100001809National Natural Science Foundation of China (NSFC) (Grants U23A20486 and 82370399).

## CRediT authorship contribution statement

**Lan Luo:** Conceptualization, Investigation, Writing – original draft. **Xiaofang Yang:** Methodology, Project administration. **Shuyuan Yi:** Investigation. **Ziyuan Dong:** Investigation. **Kan Wang:** Data curation. **Zicheng Zhu:** Investigation, Software. **Qian Gao:** Formal analysis, Validation. **Jianxue Gao:** Formal analysis. **Yu Jiang:** Validation, Visualization. **Ming Gong:** Resources, Writing – review & editing. **Hongjia Zhang:** Resources, Writing – review & editing. **Meili Wang:** Resources, Writing – review & editing. **Feilong Hei:** Funding acquisition, Supervision, Writing – review & editing.

## Declaration of competing interest

The authors declare that they have no known competing financial interests or personal relationships that could have appeared to influence the work reported in this paper.

## Data Availability

The RNA-seq data generated in this study have been deposited in the NCBI GEO database under accession code GSE287645. Additional datasets and material requests should be addressed to the corresponding author.
