# Supplementary material for: TRPV4 drives macrophage pyroptosis via mitochondrial dysfunction and mtROS-dependent NLRP3 inflammasome activation in acute lung injury
Source: Redox Biol. 2026 Jun 11;95:104255. doi: 10.1016/j.redox.2026.104255 (PMC13311294; doi:10.1016/j.redox.2026.104255)

**Supplementary Materials**

**TRPV4 drives macrophage pyroptosis via mitochondrial dysfunction and mtROS-dependent NLRP3 inflammasome activation in acute lung injury**

Lan Luo^1, 2, 3 #^, Xiaofang Yang^1 #^, Shuyuan Yi^1^, Ziyuan Dong^1^, Kan Wang^1^, Zicheng Zhu^1^, Qian Gao^1^, Jianxue Gao^1^, Yu Jiang^1^, Ming Gong^3, 4^ *, Hongjia Zhang^3, 4^ *, Meili Wang^3, 5^ *, Feilong Hei^1^ *

1. Department of Extracorporeal Circulation and Mechanical Circulation Assistants, Center for Cardiac Intensive Care, Beijing Anzhen Hospital, Capital Medical University, No. 2 Anzhen Road, Chaoyang District, Beijing 100029, China.
2. Department of Anesthesiology, Sichuan Provincial People's Hospital, School of Medicine, University of Electronic Science and Technology of China, Chengdu, 610072, China.
3. Beijing Lab for Cardiovascular Precision Medicine, Capital Medical University, Beijing, 100069, China.
4. Department of Cardiac Surgery, Beijing Anzhen Hospital, Capital Medical University, No. 2 Anzhen Road, Chaoyang District, Beijing 100029, China.
5. Department of Physiology and Pathophysiology, School of Basic Medical Sciences, Capital Medical University, Beijing, 100069, China.

# These authors have contributed equally to this work

* *Correspondence to*:

Feilong Hei, M.D., E-mail: heifeilong@mail.ccmu.edu.cn

Meili Wang, Ph.D., E-mail: [wangmeili@ccmu.edu.cn](mailto:wangmeili@ccmu.edu.cn)

Hongjia Zhang, M.D., E-mail: zhanghongjia722@ccmu.edu.cn

Ming Gong, M.D., E-mail: [gongming@mail.ccmu.edu.cn](mailto:gongming@mail.ccmu.edu.cn)


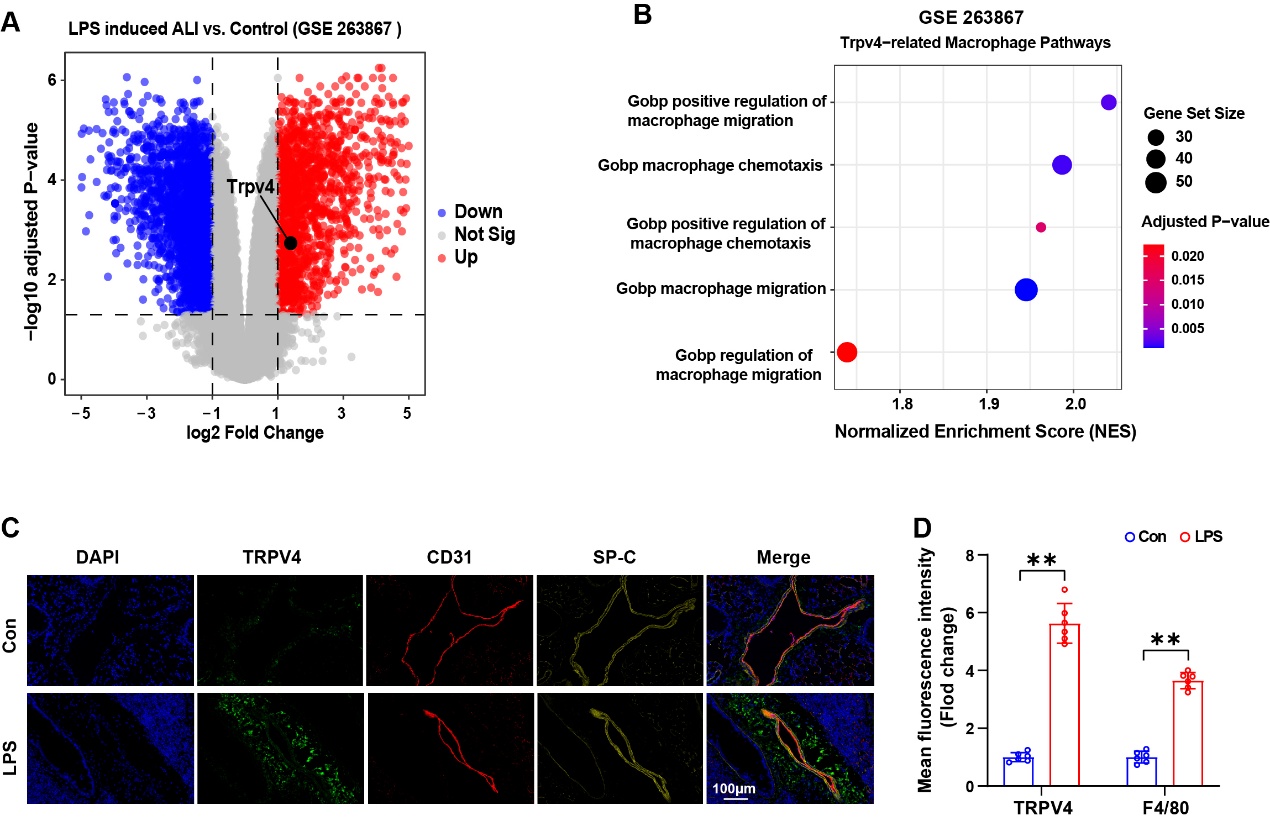
**Figure S1 Transcriptomic analysis and cellular localization of TRPV4 in LPS-induced acute lung injury**

A, Volcano plot illustrating differentially expressed genes in the GSE263867 dataset. B, Gene Ontology (GO) enrichment analysis of differentially expressed genes identified from the GSE263867 dataset. C, Tyramide signal amplification (TSA)-based immunofluorescence staining showing the localization of TRPV4 with CD31 and SP-C in the lung sections from mice treated with PBS or LPS. Scale bar: 100μm. D, Quantification of mean fluorescence intensity of TRPV4 and F4/80 in the lung sections from mice treated with PBS or LPS. n=6. Student’s t-test. All data are mean ± SD. ^*^*P* < 0.05 vs. Con group, ^**^*P* < 0.01 vs. Con group.


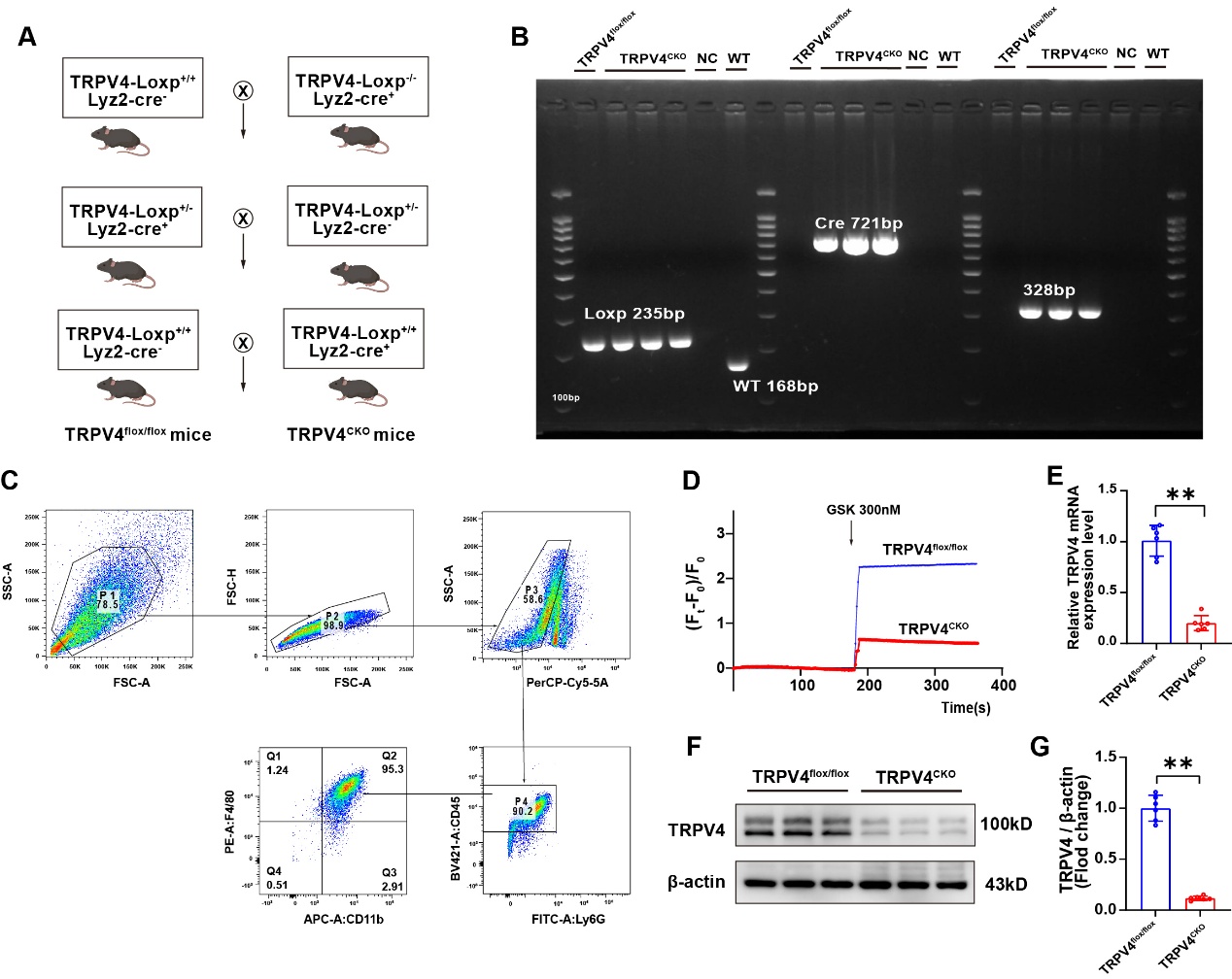


**Figure S2 Generation and validation of myeloid-specific TRPV4 conditional knockout mice**

A, Breeding scheme for generating TRPV4^flox/flox^ and TRPV4^CKO^ mice. B, Representative PCR genotyping results of TRPV4^flox/flox^ and TRPV4^CKO^ mice. C, Flow cytometry gating strategy used to identify BMDMs. D, Calcium imaging analysis of BMDMs from TRPV4^flox/flox^ and TRPV4^CKO^ mice. E, RT-qPCR analysis of TRPV4 mRNA expression in the lung tissues of TRPV4^flox/flox^ and TRPV4^CKO^ mice. n=6. F-G Western blot and quantification of TRPV4 protein level in the lungs of BMDM from TRPV4^flox/flox^ and TRPV4^CKO^ mice. n=6. All data are mean ± SD. All data are mean ± SD. Statistical significance was determined by Student’s t-test. ^*^*P* < 0.05 vs. TRPV4^flox/flox^ group, ^**^*P* < 0.01 vs. TRPV4^flox/flox^ group.


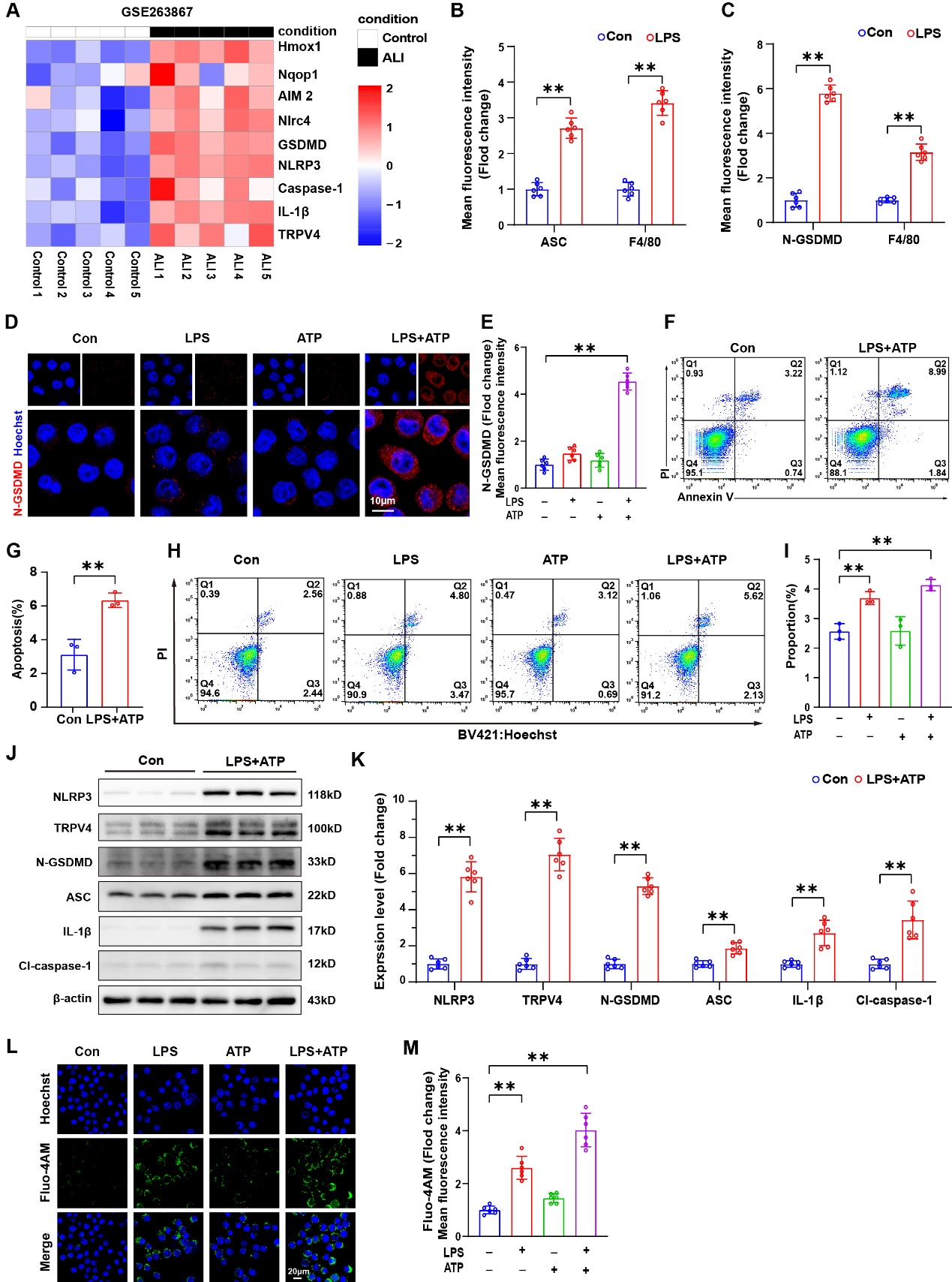
**Figure S3 Identification and validation of NLRP3 inflammasome activation and pyroptosis in macrophages during LPS-induced lung injury**

A, Transcriptomic profiling highlights activation of the NLRP3 inflammasome pathway in ALI. Heatmap showing the expression patterns of genes associated with the NLRP3 signaling pathway in the GSE263867 dataset. B–C, Inflammasome activation in pulmonary macrophages in vivo. Quantification of mean fluorescence intensity of ASC (B) and N-GSDMD (C) in F4/80-positive macrophages in lung sections from mice treated with PBS or LPS. n = 6. D–I, LPS+ATP induces inflammasome activation and pyroptotic cell death in macrophages *in vitro*. Representative images and quantification of N-GSDMD expression (D–E). Flow cytometric assessment and quantification of the percentage of PI^+^/Annexin^+^ macrophages (F–G) and PI^+^/Hoechst^+^ macrophages (H–I). n = 6 (D–E), n = 3 (F–I). J–K, Western blot analysis and quantification of TRPV4, NLRP3, ASC, Cl-caspase-1, IL-1β, and N-GSDMD in BMDMs treated with PBS or LPS. n = 6. L-M, Intracellular Ca²⁺ accumulation in macrophages following LPS+ATP stimulation, assessed by Fluo-4 AM staining. Scale bar: 20 μm. n = 6. All data are mean ± SD. Statistical significance was determined by one-way ANOVA followed by Tukey’s post-hoc test. ^*^*P* < 0.05, ^**^*P* < 0.01 vs. Con group.


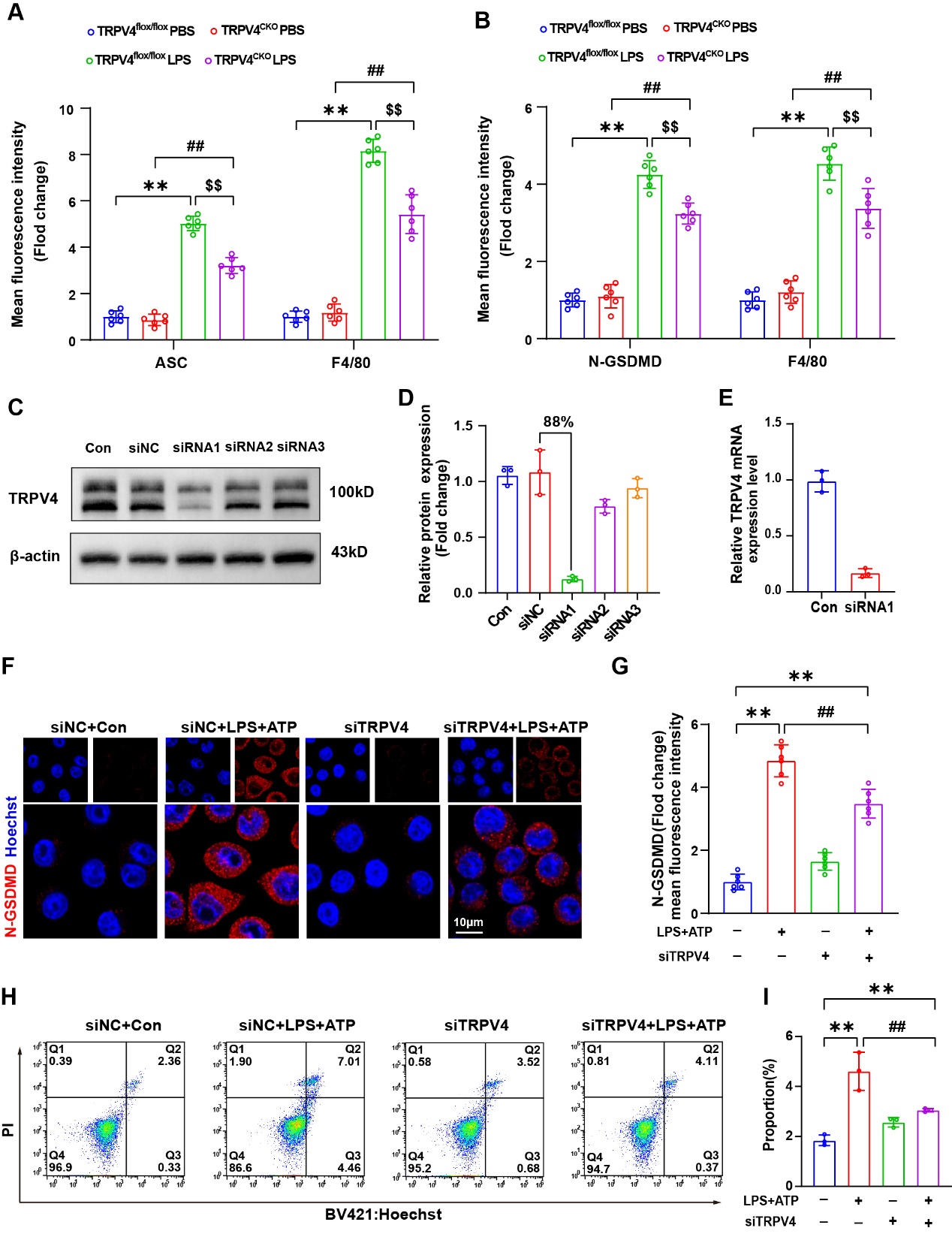


**Figure S4 Deletion or knockdown of TRPV4 inhibits NLRP3 inflammasome activation and pyroptosis in macrophages**

A–B, TRPV4 deficiency attenuates inflammasome activation in pulmonary macrophages in vivo. Quantification of the mean fluorescence intensity of ASC (A) and N-GSDMD (B) in F4/80-positive macrophages in lung sections from TRPV4^flox/flox^ and TRPV4^CKO^ mice treated with PBS or LPS. n = 6. C–E, Validation of TRPV4 knockdown efficiency in macrophages. Western blot analysis and quantification of TRPV4 protein levels following transfection with negative control siRNA (siNC) or three independent TRPV4-targeting siRNAs (siRNA1–3) (C–D), and RT-qPCR analysis of TRPV4 mRNA expression (E). n = 3. F–I, TRPV4 silencing suppresses pyroptosis in macrophages. Representative images and quantification of N-GSDMD expression (F–G), and flow cytometric assessment and quantification of the percentage of PI^+^/Hoechst^+^ in macrophages transfected with siTRPV4 prior to LPS+ATP stimulation (H–I). n = 6 (F–G), n = 3 (H–I). Data are presented as mean ± SD. Statistical significance was determined using one-way ANOVA followed by Tukey’s post-hoc test. ^*^*P* < 0.05, ^**^*P* < 0.01 vs. TRPV4^flox/flox^ PBS or siNC + DMSO group; ^#^*P* < 0.05, ^##^*P* < 0.01 vs. TRPV4^CKO^ PBS or siNC + LPS + ATP group; ^$^*P* < 0.05, ^$$^*P* < 0.01 vs. TRPV4^flox/flox^ LPS group.


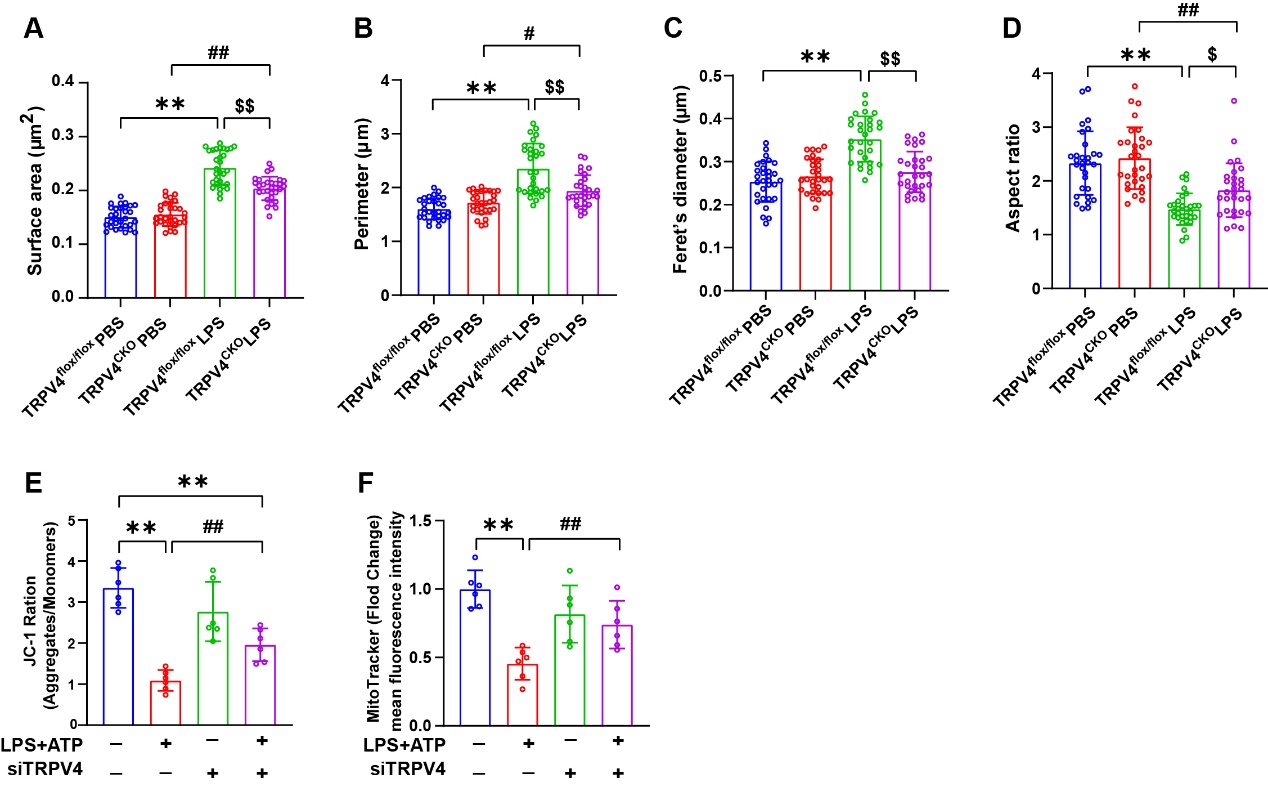


**Figure S5 TRPV4 knockdown restores mitochondrial membrane potential and reduces aberrant mitochondria in macrophages under inflammatory conditions**

A-D, Quantitative analysis of mitochondrial morphological parameters, including mitochondrial surface area, perimeter, Feret’s diameter, and aspect ratio. Thirty mitochondria were analyzed per group. E, Quantification of red-to-green fluorescence ratio in JC-1 staining of macrophages transfected with siTRPV4 subsequently stimulated with LPS plus ATP. n=6. F, Quantification of the mean fluorescence intensity in MitoTracker staining of macrophages transfected with siTRPV4 followed by stimulation with LPS+ATP. n=6. All data are mean ± SD. significance was determined using one-way ANOVA followed by Tukey’s post-hoc test. ^*^*P* < 0.05, ^**^*P* < 0.01 vs. TRPV4^flox/flox^ PBS or siNC + DMSO group; ^#^*P* < 0.05, ^##^*P* < 0.01 vs. TRPV4^CKO^ PBS or siNC + LPS + ATP group; ^$^*P* < 0.05, ^$$^*P* < 0.01 vs. TRPV4^flox/flox^ LPS group.

**
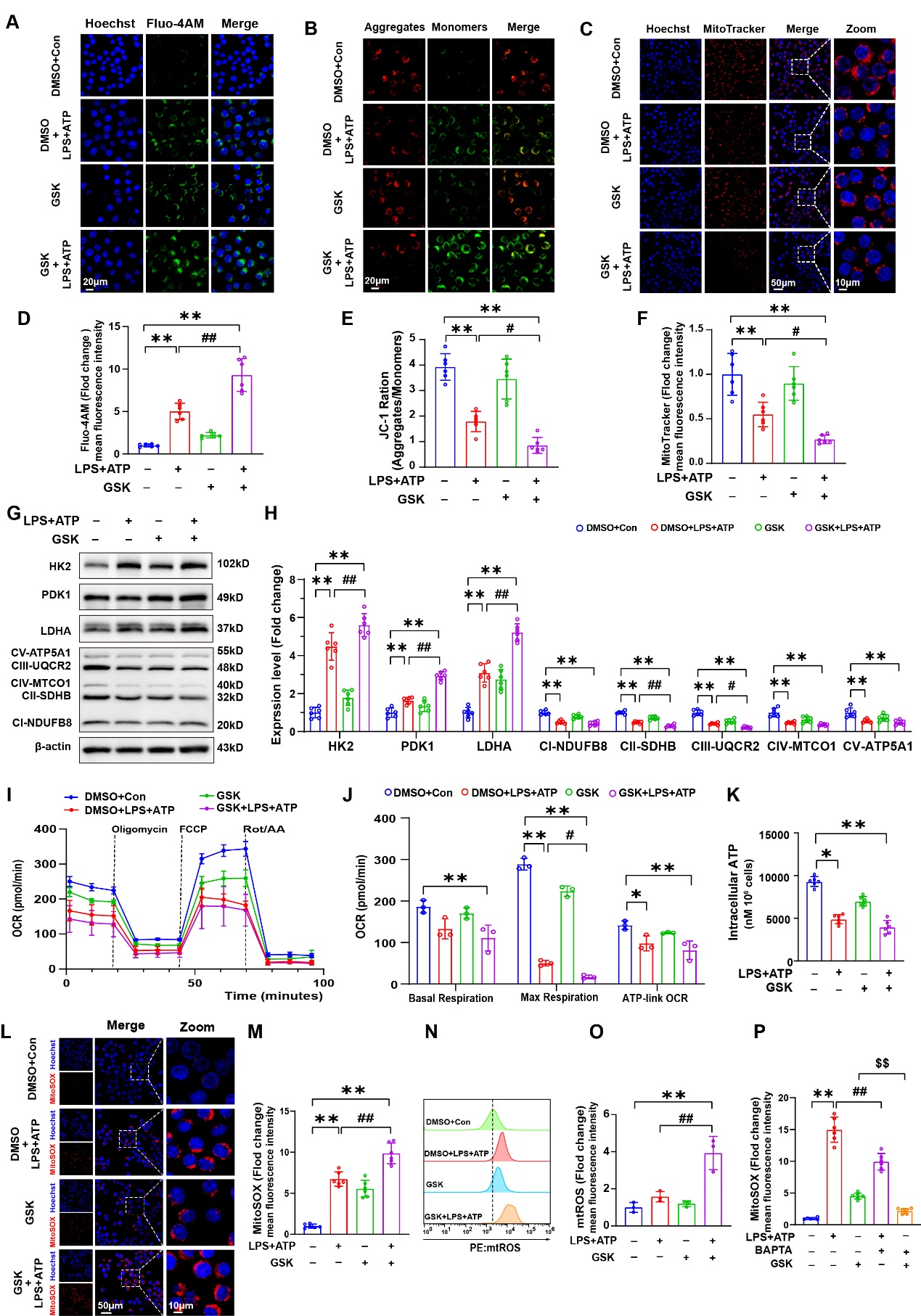
**

**Figure S6 TRPV4 activation induces mitochondrial dysfunction and mtROS accumulation, promoting NLRP3 inflammasome activation and macrophage pyroptosis**

A–C, TRPV4 activation induces Ca²⁺ influx and mitochondrial dysfunction in macrophages. Representative images of Fluo-4 AM staining (A), JC-1 staining (B), and MitoTracker staining (C) in macrophages pretreated with the TRPV4 agonist GSK followed by LPS+ATP stimulation. Scale bars: 20 μm (A–B); 50 μm and 10 μm (insets, C). D–F, Quantification of intracellular Ca²⁺ levels (D), mitochondrial membrane potential (JC-1 red/green ratio) (E), and mitochondrial mass (F) in macrophages from the indicated groups. n = 6. G–K, TRPV4 activation disrupts mitochondrial bioenergetics. Western blot analysis and quantification of OXPHOS-related proteins (ATP5A1, MTCO1, UQCRC2, SDHB, NDUFB8) and glycolytic enzymes (HK2, PDK1, LDHA) (G-H), Seahorse analysis and quantification of OCR (I–J), and intracellular ATP levels (K). n = 6 (H, K), n = 3 (J). L–O, TRPV4 activation promotes mtROS accumulation. Representative MitoSOX staining (L–M) and flow cytometric analysis of mtROS levels (N), with quantification (O). Scale bars: 50 μm and 10 μm (insets). n = 6 (M), n = 3 (O). P, Quantification of MitoSOX staining in macrophages pretreated with or without BAPTA or GSK, followed by LPS+ATP stimulation. n = 6. Data are presented as mean ± SD. Statistical significance was determined using one-way ANOVA followed by Tukey’s post-hoc test. ^*^*P* < 0.05, ^**^*P* < 0.01 vs. DMSO + Con group; ^#^*P* < 0.05, ^##^*P* < 0.01 vs. DMSO + LPS + ATP group. ^$^*P* < 0.05, ^$$^*P* < 0.01 vs. GSK group.


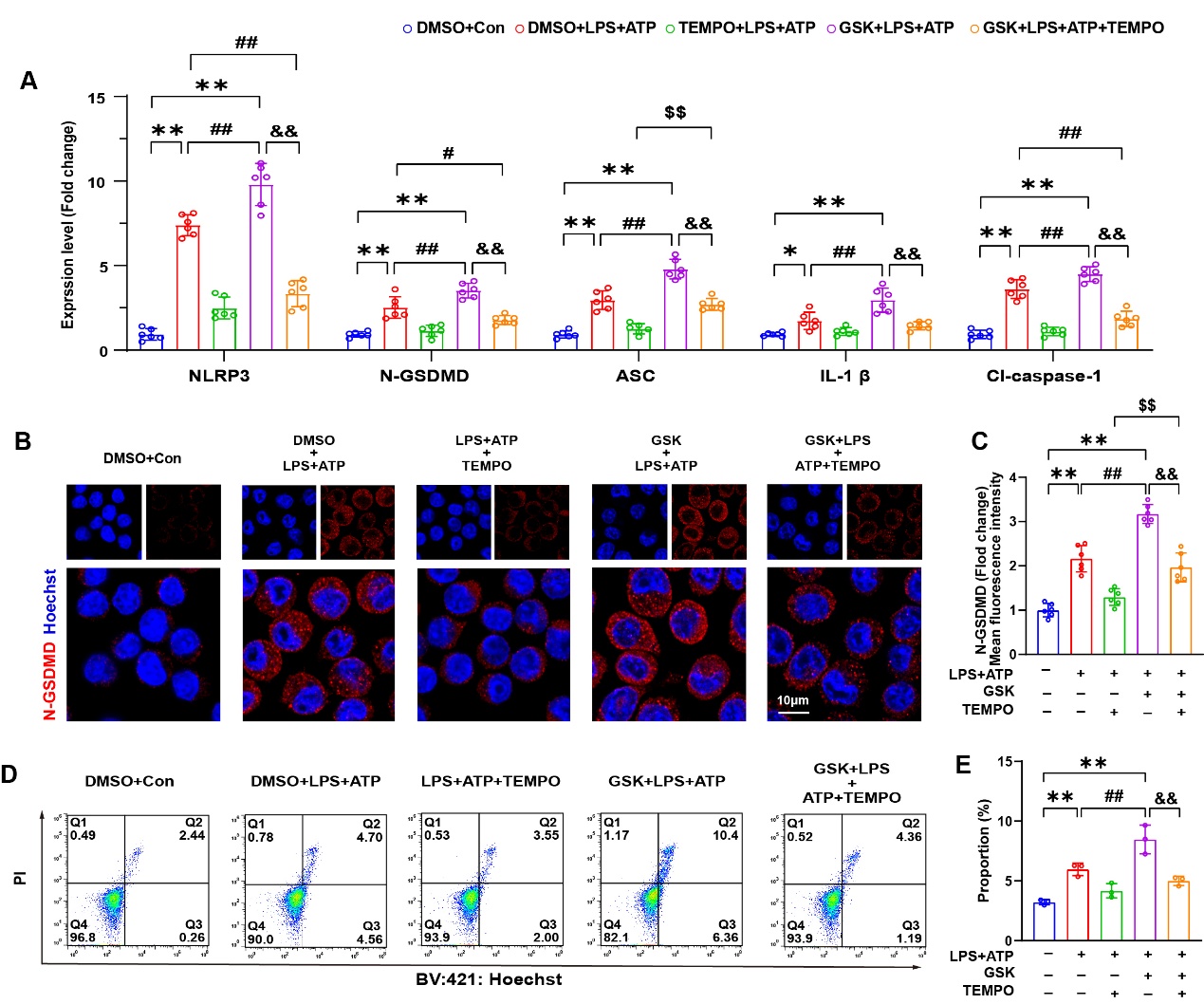
**Figure S7 TRPV4-dependent mtROS production is essential for NLRP3 inflammasome activation and pyroptosis in macrophages**

A–E, mtROS scavenging attenuates TRPV4-driven inflammasome activation and pyroptosis in macrophages. Western blot analysis and quantification of NLRP3, Cl-caspase-1, IL-1β, ASC, and N-GSDMD protein levels (A), representative images and quantification of N-GSDMD expression (B–C), and flow cytometric assessment and quantification of the percentage of PI^+^/Hoechst^+^ macrophages pretreated with the TRPV4 agonist GSK in the presence or absence of the mitochondrial ROS scavenger TEMPO, followed by LPS+ATP stimulation (D–E). n = 6 (A–C), n = 3 (D–E). Data are presented as mean ± SD. Statistical significance was determined using one-way ANOVA followed by Tukey’s post-hoc test. ^*^*P* < 0.05, ^**^*P* < 0.01 vs. DMSO + Con group; ^#^*P* < 0.05, ^##^*P* < 0.01 vs. DMSO + LPS + ATP group; ^$^*P* < 0.05, ^$$^*P* < 0.01 vs. TEMPO + LPS + ATP group; ^&^*P* < 0.05, ^&&^*P* < 0.01 vs. GSK + LPS + ATP group.

**
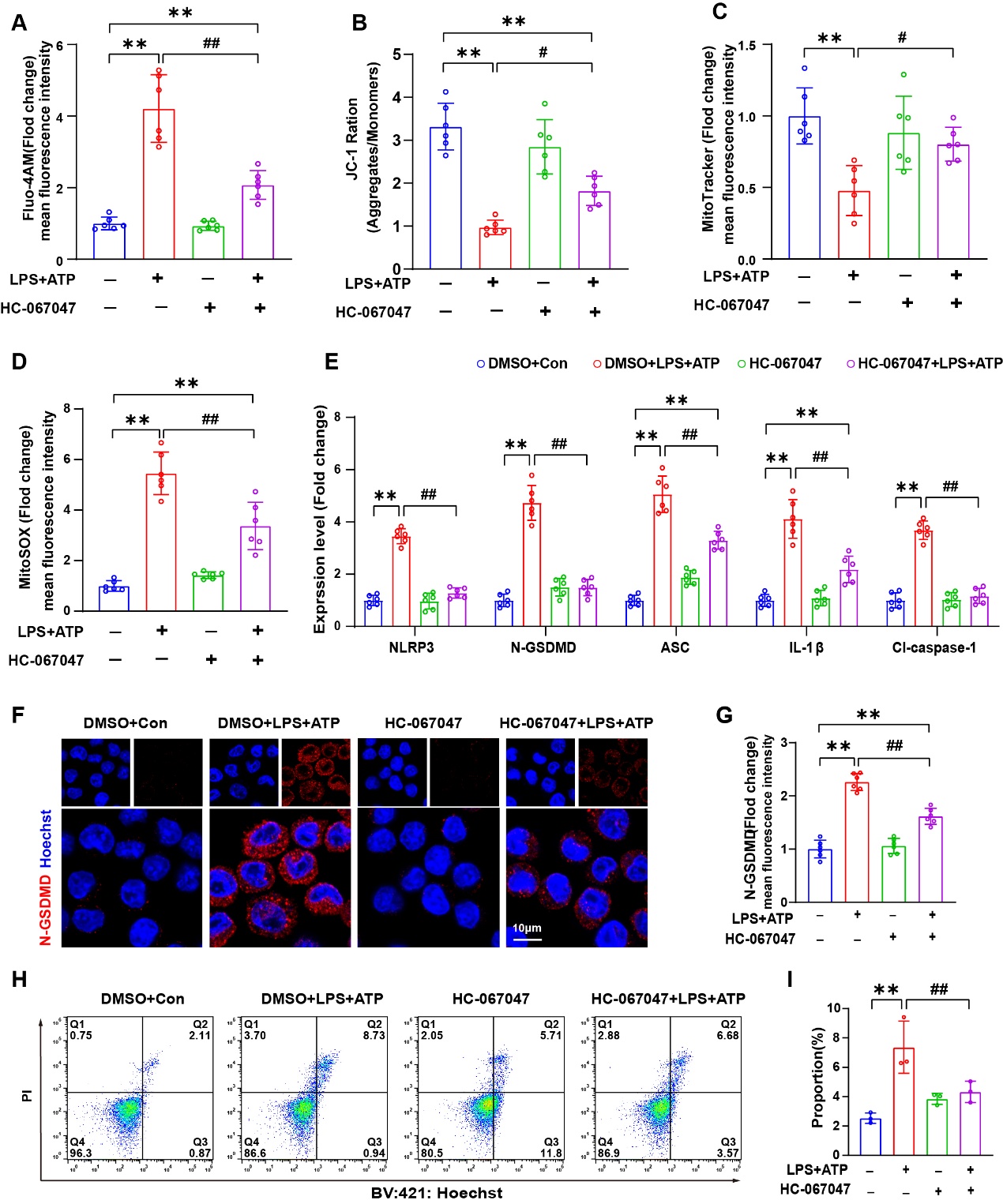
**

**Figure S8 Pharmacological blockade of TRPV4 alleviates mitochondrial dysfunction, NLRP3 inflammasome activation, and pyroptosis in macrophages**

A–D, TRPV4 inhibition suppresses Ca^2+^ influx, mitochondrial dysfunction, and mtROS production in macrophages. Quantification of Fluo-4 AM fluorescence intensity (A), JC-1 red/green fluorescence ratio (B), MitoTracker fluorescence intensity (C), and MitoSOX fluorescence intensity (D) in macrophages pretreated with the TRPV4 antagonist HC-067047 followed by LPS+ATP stimulation. n = 6. E–G, TRPV4 inhibition attenuates NLRP3 inflammasome activation and pyroptosis. Western blot analysis and quantification of NLRP3, Cl-caspase-1, IL-1β, ASC, and N-GSDMD protein levels (E), representative images and quantification of N-GSDMD expression (F–G) in macrophages from the indicated groups. n = 6. H-I, Flow cytometric assessment and quantification of the percentage of PI^+^/Hoechst^+^ macrophages pretreated with HC-067047 followed by LPS+ATP stimulation. n = 3. Data are presented as mean ± SD. Statistical significance was determined using one-way ANOVA followed by Tukey’s post hoc test. ^*^*P* < 0.05, ^**^*P* < 0.01 vs. DMSO + Con group; ^#^*P* < 0.05, ^##^*P* < 0.01 vs. DMSO + LPS + ATP group.


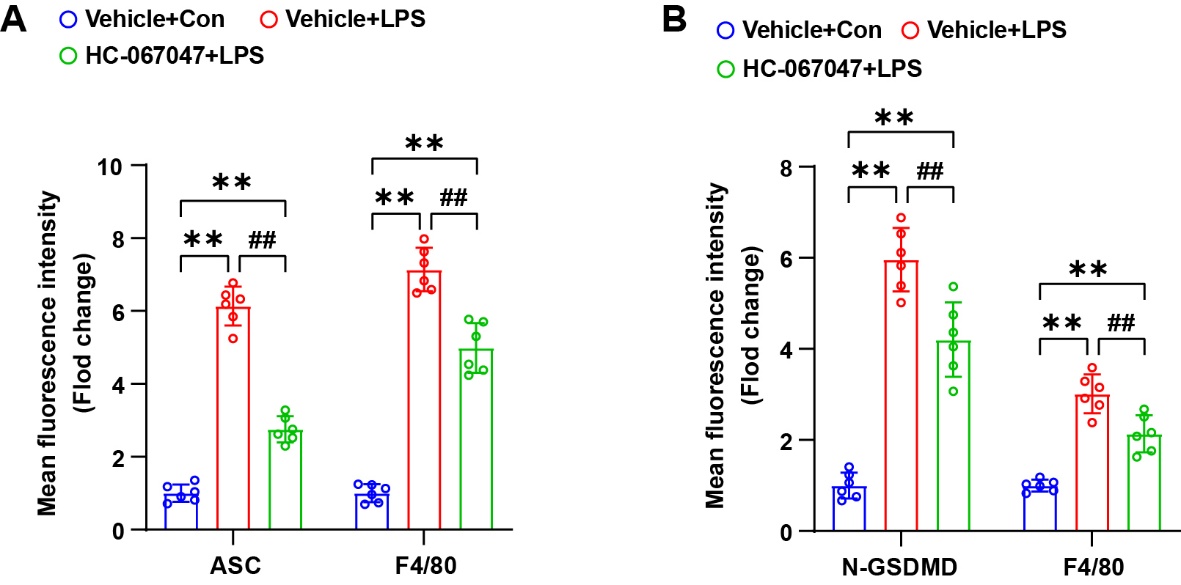


**Figure S9 TRPV4 antagonist HC-067047 suppresses the expression of ASC and N-GSDMD in lung macrophages of LPS-treated mice**

A–B, TRPV4 inhibition attenuates inflammasome activation in pulmonary macrophages in vivo. Quantification of the mean fluorescence intensity of ASC (A) and N-GSDMD (B) in F4/80-positive macrophages in lung sections from mice treated with vehicle or HC-067047 followed by PBS or LPS. n = 6. Data are presented as mean ± SD. Statistical significance was determined using one-way ANOVA followed by Tukey’s post-hoc test. ^*^*P* < 0.05, ^**^*P* < 0.01 vs. Vehicle + Con group; ^#^*P* < 0.05, ^##^*P* < 0.01 vs. Vehicle + LPS group.


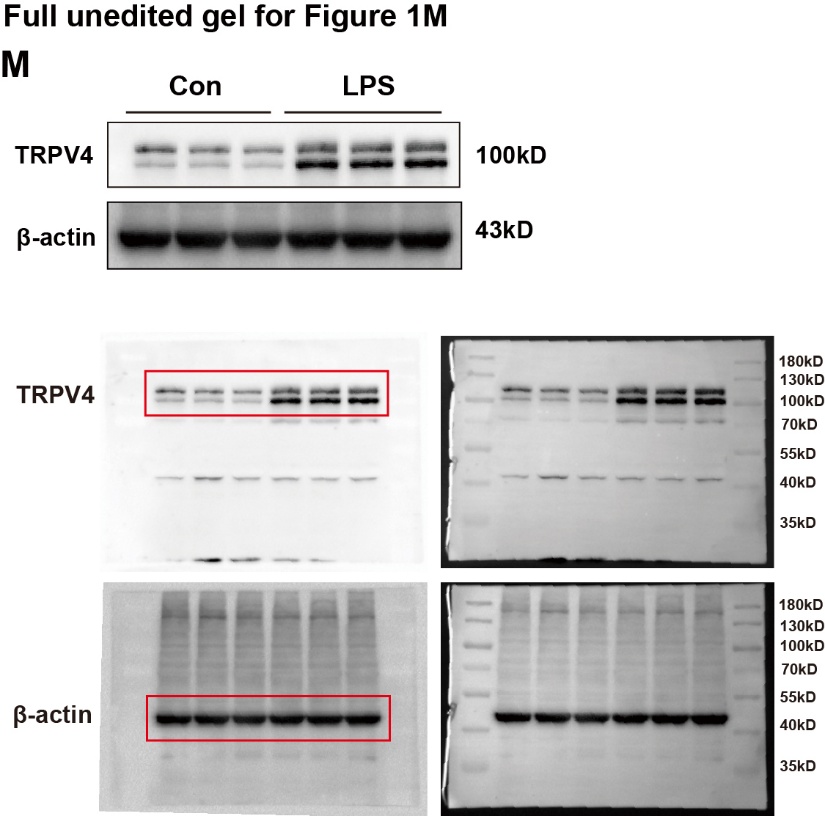


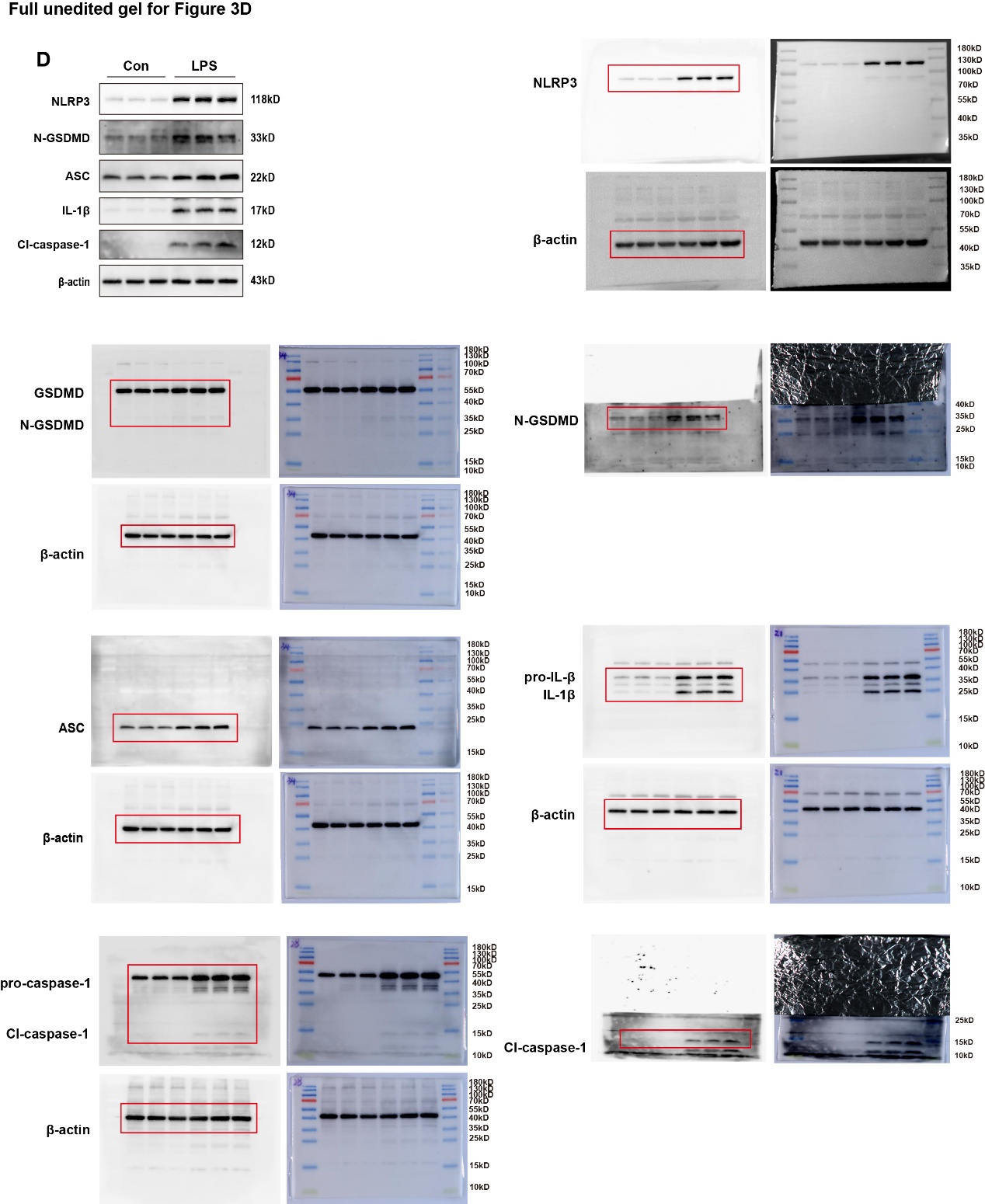


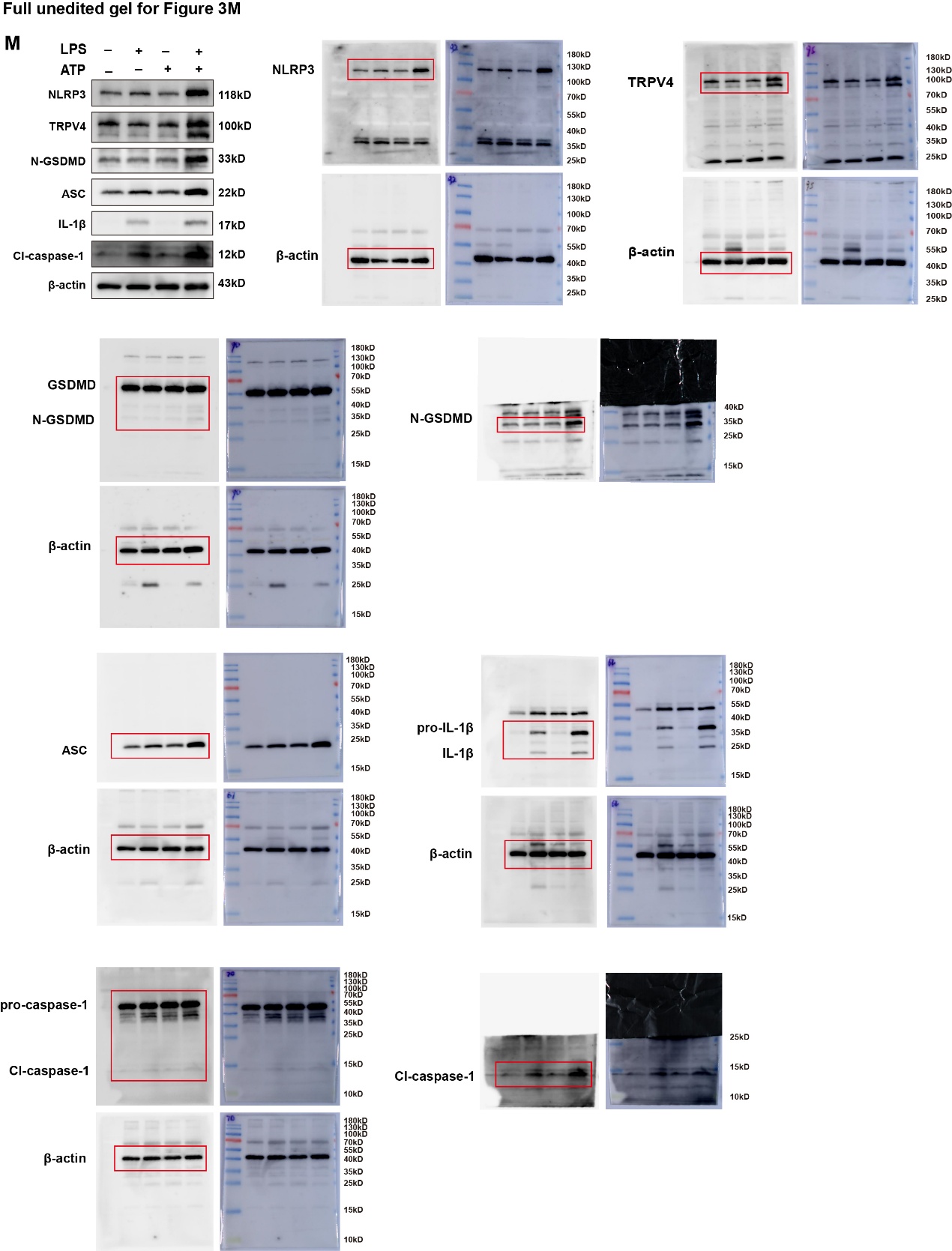


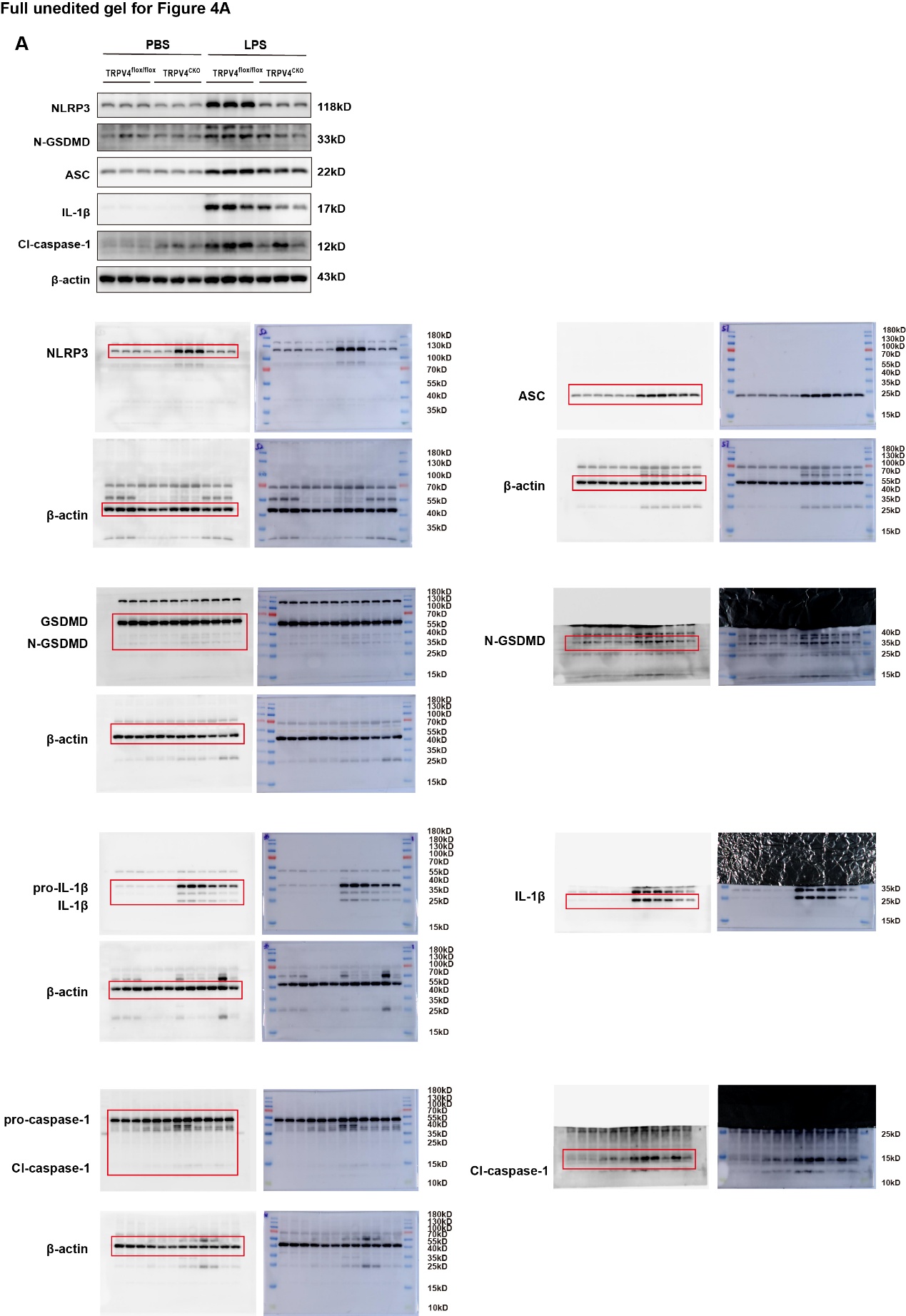


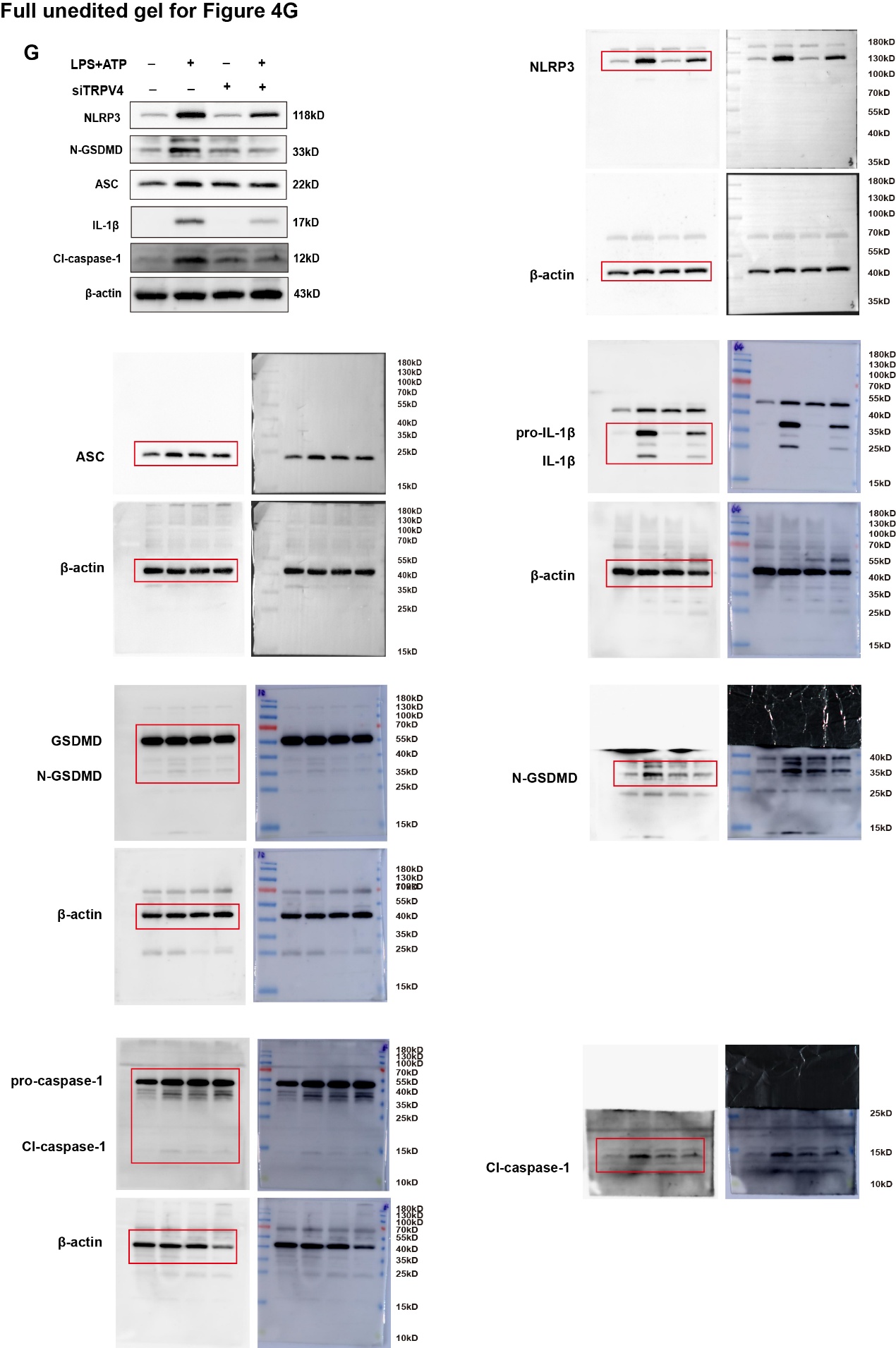


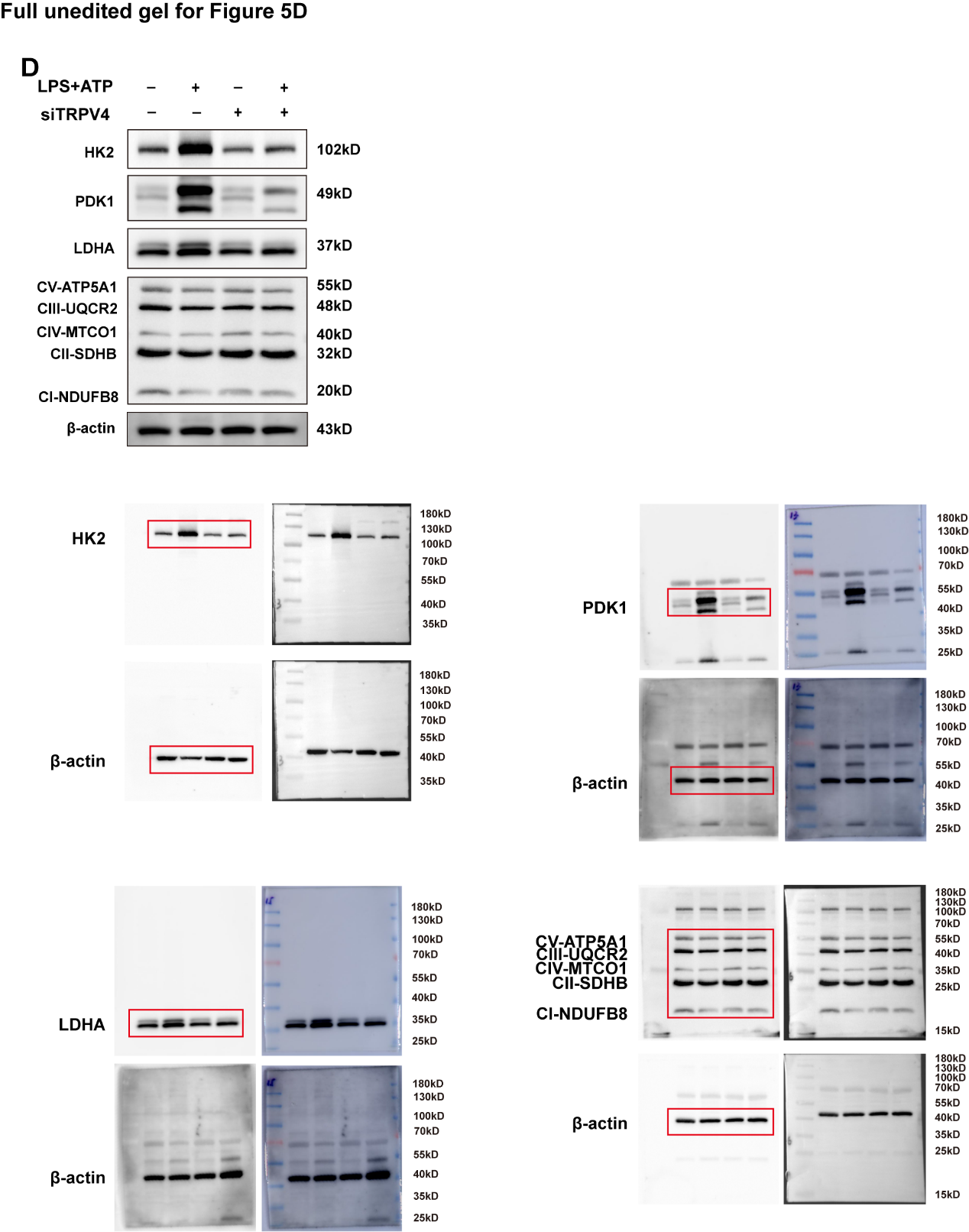


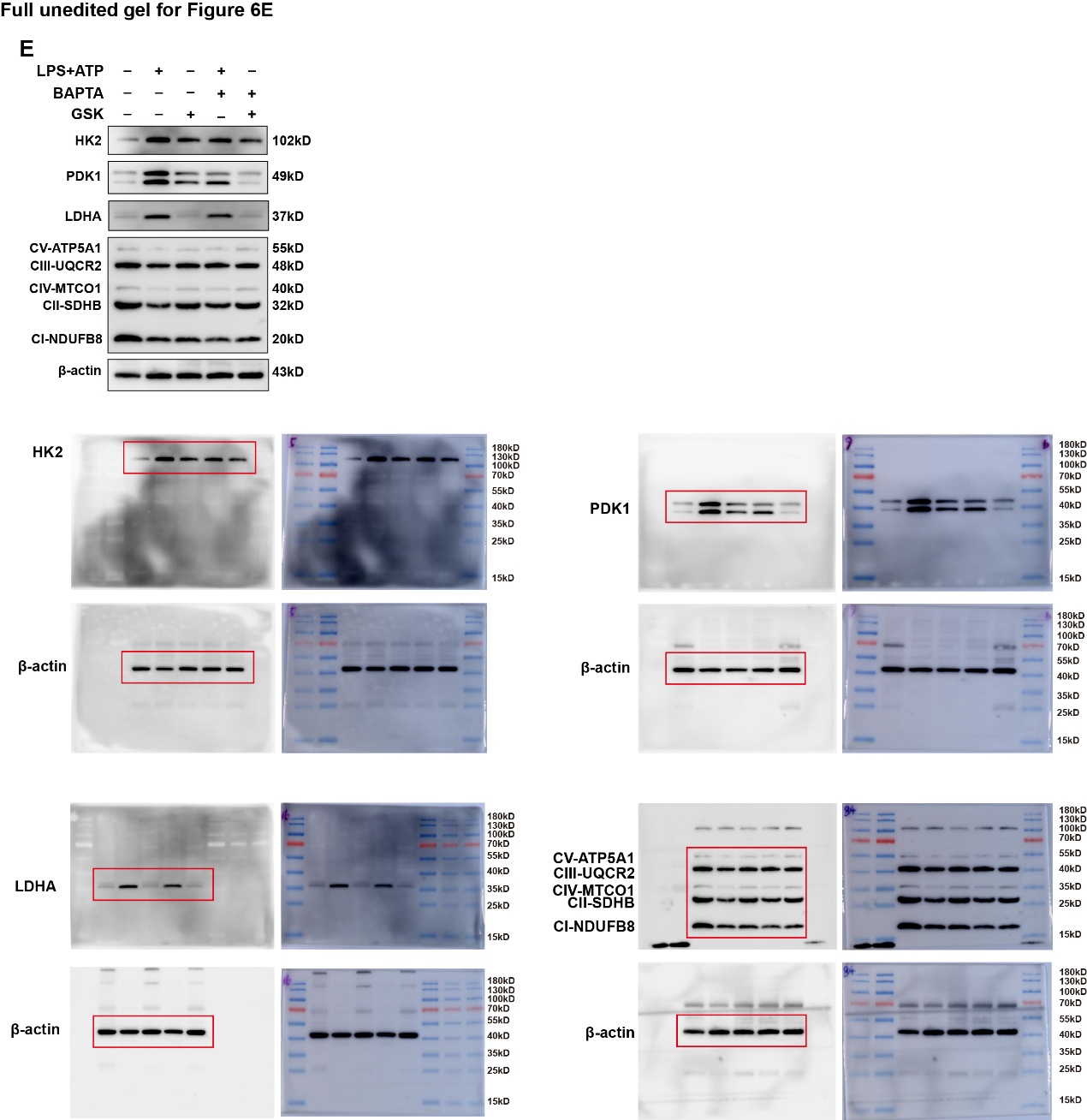


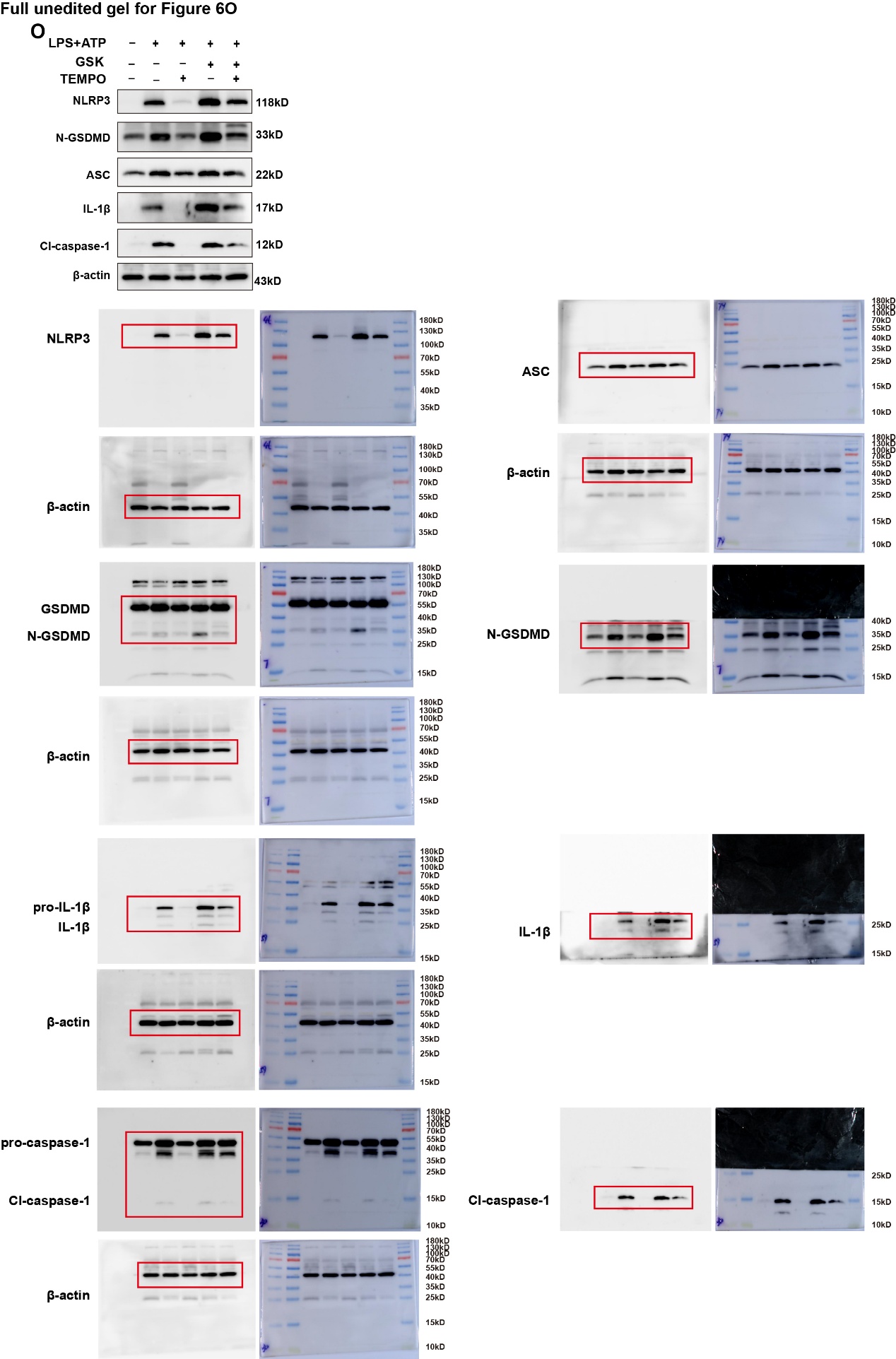


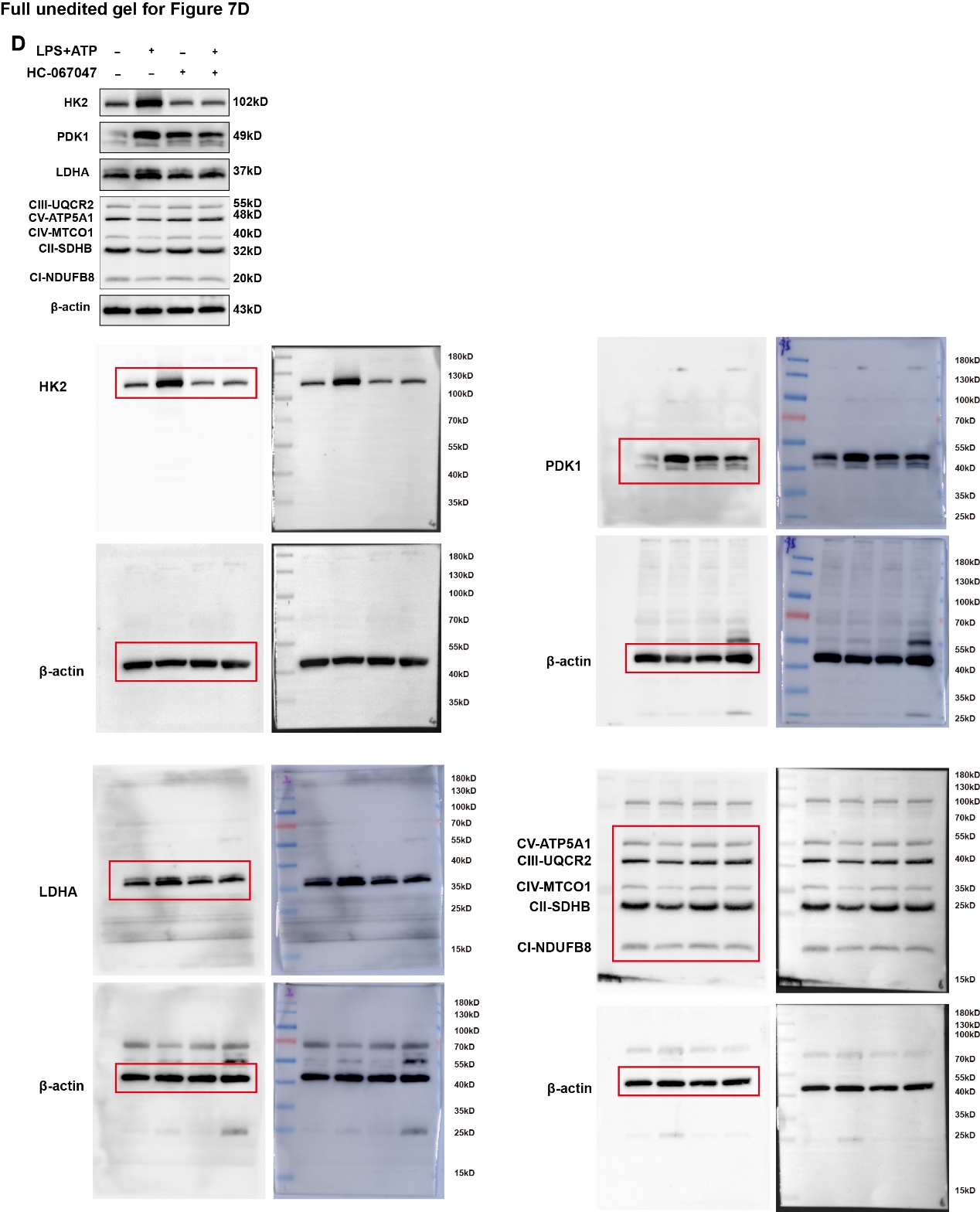


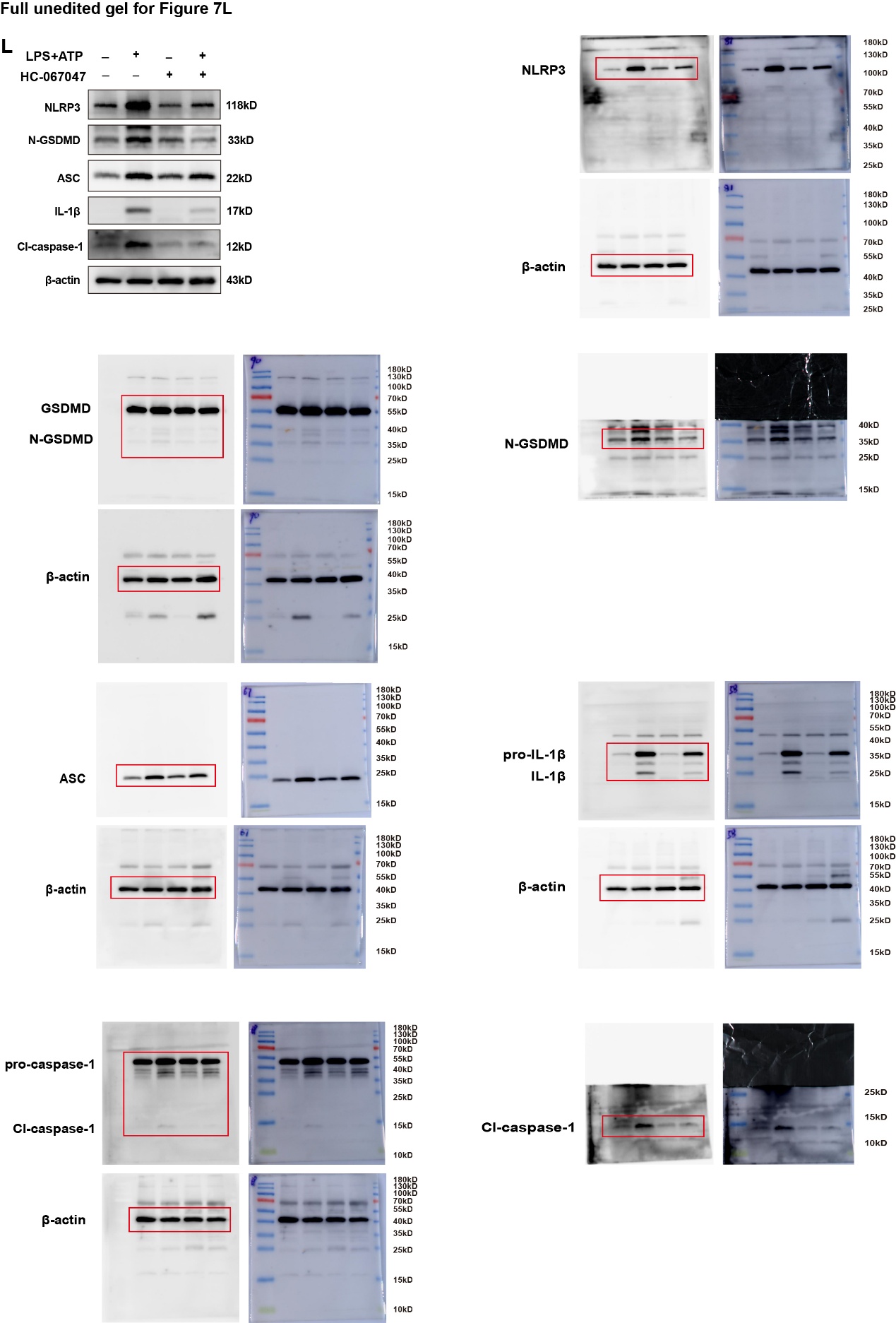


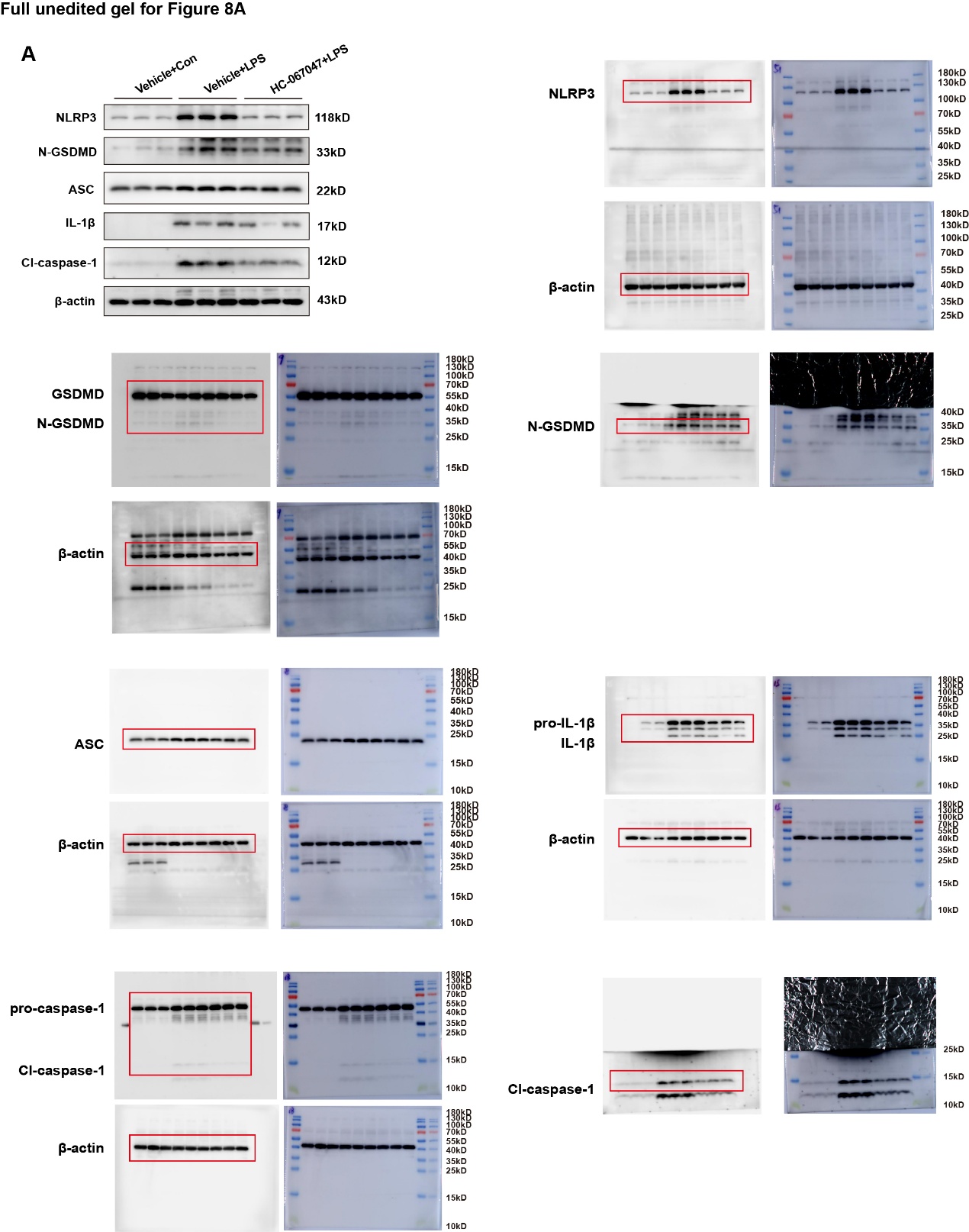


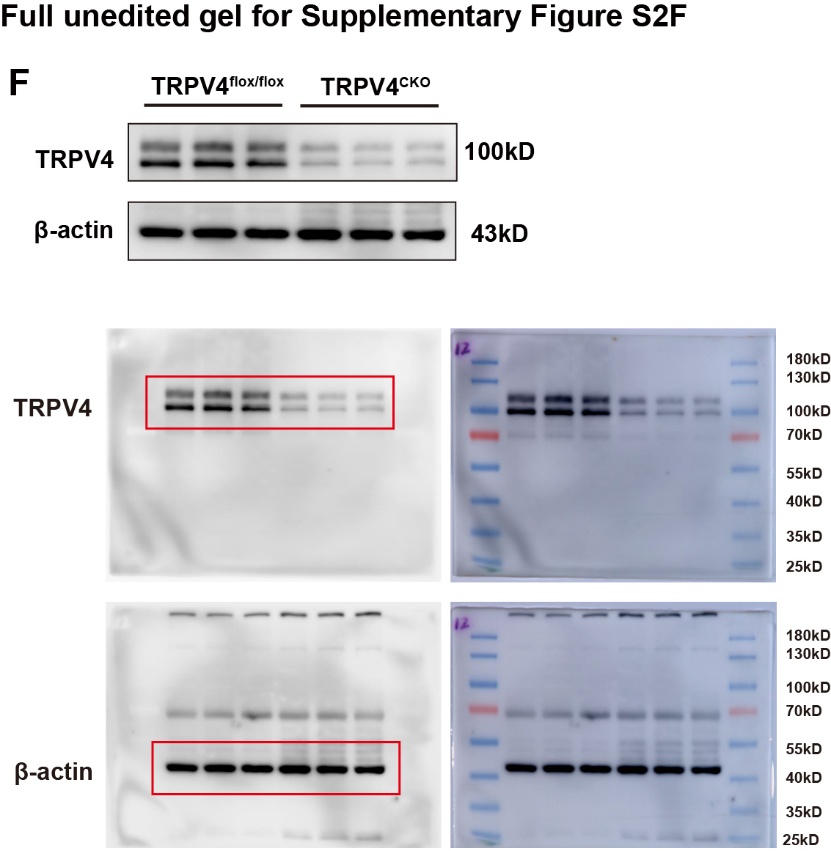


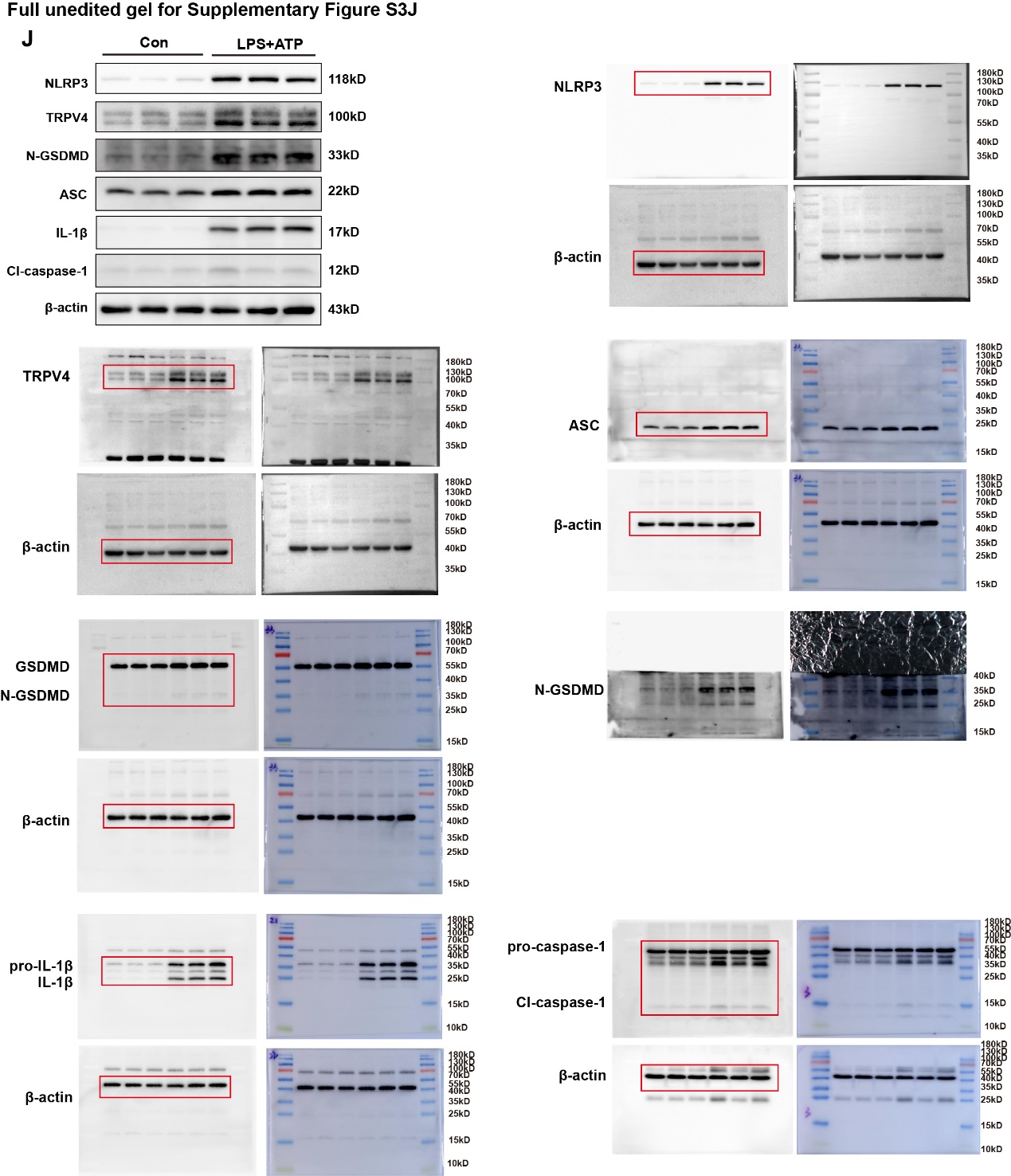


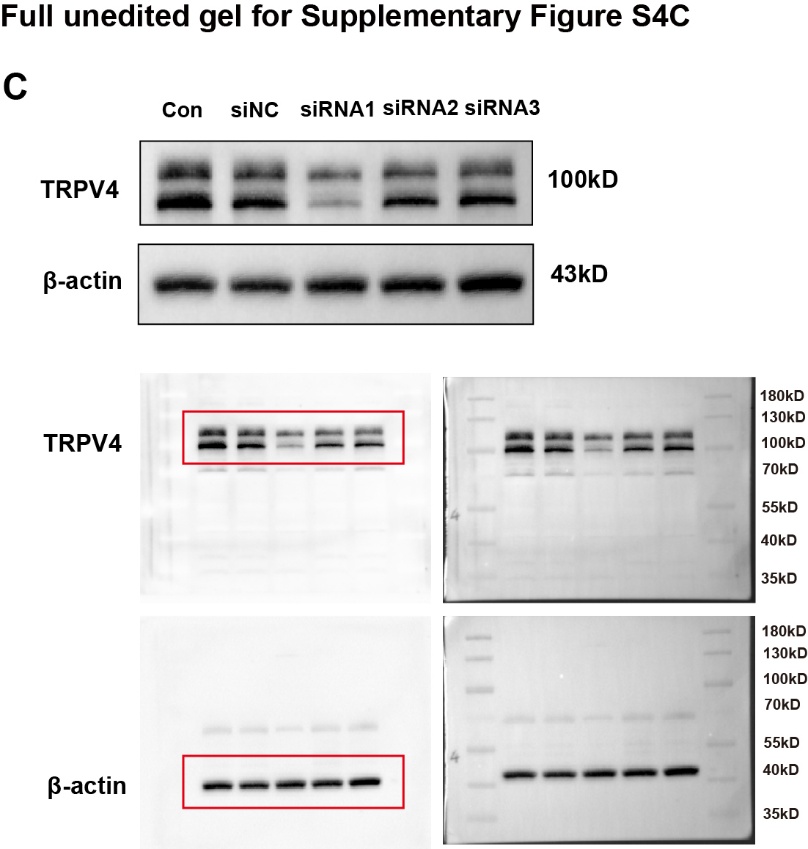


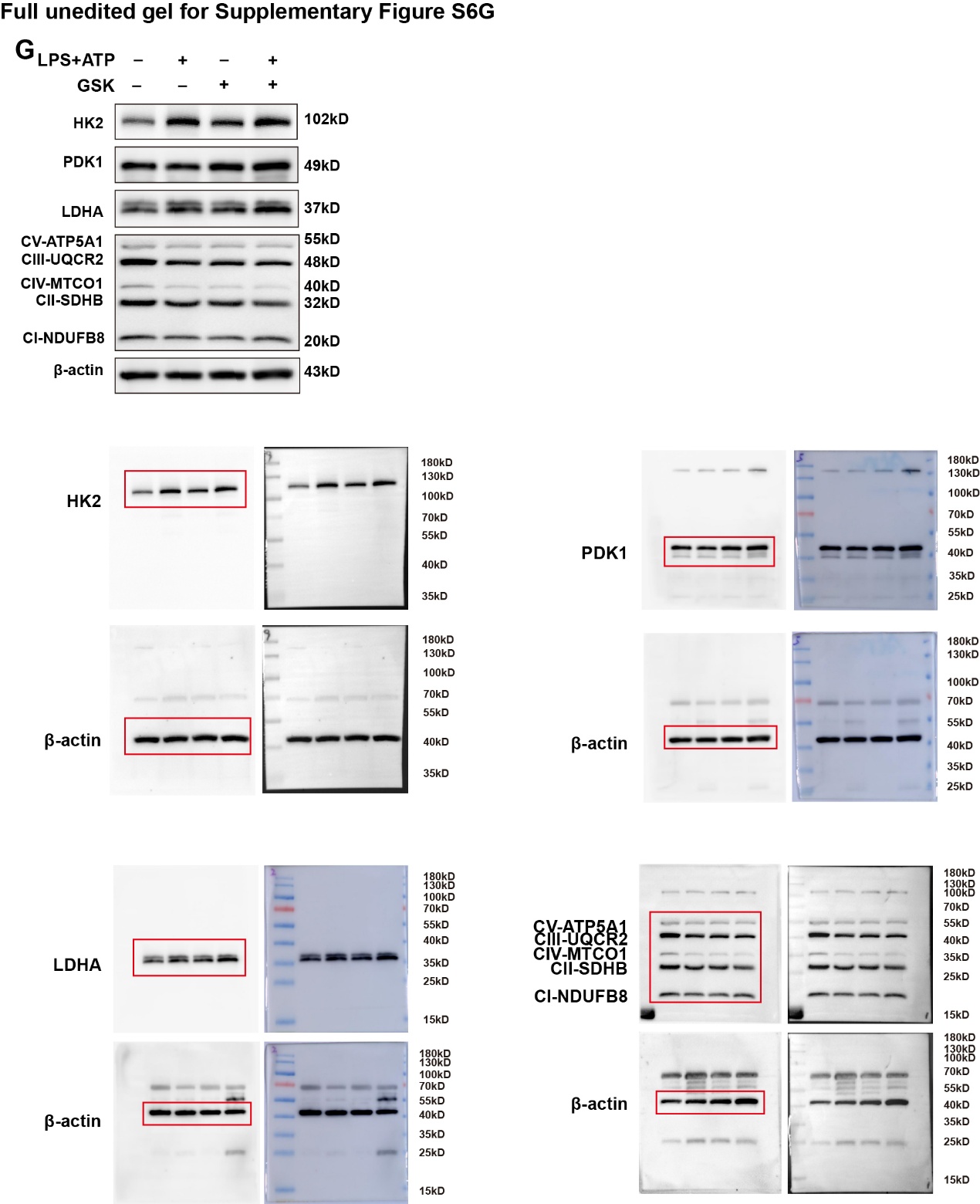

Supplement: Multimedia component 1 [file mmc1.docx]
